# Supplementary material for: Area level impacts on emergency hospital admissions of the integrated care and support pioneer programme in England: difference-in-differences analysis
Source: BMJ Open. 2019 Aug 18;9(8):e026509. doi: 10.1136/bmjopen-2018-026509 (PMC6701574; doi:10.1136/bmjopen-2018-026509)
Supplement: Supplementary data [file bmjopen-2018-026509supp001.pdf]

SUPPLEMENTARY MATERIAL

1. Target populations, integration mechanisms, activities and emergency admission focus of Wave 1 Pioneers
2. Local Authority to Pioneer Lookup
3. Emergency admission rates for individual local authorities within Pioneers
4. Sensitivity analysis: excluding Stoke and North Staffordshire Pioneer
5. Sensitivity analysis: individual baseline years compared to 2014/15

Keeble E, *et al. BMJ Open* 2019; 9:e026509. doi: 10.1136/bmjopen-2018-026509

1. Target populations, integration mechanisms, activities and emergency admission focus of Wave 1 Pioneers

Pioneer	Target Population(s)	Integration Mechanisms	Examples of Specific Activities	Reduced emergency admissions
Barnsley	Whole population with focus on children and families	Integrated programme boards;<br>Adopting a citizenship approach at all levels of the community to move from intervention to prevention;<br>Provision of information, advice, and signposting through services such as telecare.	Universal information and advice service;<br>Be Well Barnsley: community orientated prevention;<br>Immediate care review;<br>Integrated personal budgets	Aim
Cheshire	Older adults with chronic conditions; individuals with mental health issues; complex needs families	Integration commissioning through a joint governance board, redesigned care and intervention pathways and joint investment plan involving the voluntary and community sectors;<br>Integration of care management and investment into integration enablers, such as shared records and telehealth services.	New funding/contracting model for the acute sector and community care;<br>Hospital at home;<br>Partnership working between Health Care Management Financial Association, Health Care Services and Net Orange to reduce hospital admissions;	Outcome
Cornwall and Isles of Scilly	People who are at risk of becoming high users of health and social care services	Integrated leadership through shared goals and performance measures, information sharing and a new funding contract;<br>Investment in telehealth;<br>Integrated care through multi-disciplinary teams, workforce development, information sharing;<br>Better prevention through early risk identification and management of acute care.	Personal health budgets;<br>Specific acute activities:<br>- Rapid assessment upon presentation to acute care<br>- Discharge support<br>- Visual ward model	Outcome

Keeble E, *et al. BMJ Open* 2019; 9:e026509. doi: 10.1136/bmjopen-2018-026509

Greenwich	Older people with complex or chronic conditions; individuals with mental health issues	Integrated commissioning through joint health and social care assessment, planning and management;<br>Integrated care through multi-disciplinary teams including the voluntary sector, shared care plans, information and investment in self-care initiatives such as telehealth;<br>Better prevention and management of acute care.	Personal health budgets;<br>Specific acute activities:<br>- Risk stratification to identify 11.5% at highest risk of hospital admission within one year<br>- Virtual admission avoidance team<br>- Greenwich Joint Emergency Team (JET)<br>- Access to the medical diagnostic centre at the acute trust and outreach specialist opinion<br>- Hospital Intervention Discharge Team<br>- Community Assessment and Rehabilitation Teams (CARs).	Outcome
Islington	Whole population with focus on vulnerable older people;<br>people with long term conditions;<br>young people at risk;<br>people with mental health issues.	Integrated commissioning including patients through shared vision, planning, and information sharing;<br>Integrated care activities including information sharing;<br>Better prevention activities, including self-management support through personal health budgets and telehealth.	Incentivising acute and community healthcare services through CQUIN<br>Care pathways for conditions like COPD.	Aim
Kent	Adults with long term conditions and older people	Integrated commissioning through information sharing and data mapping;<br>Integrated care through multidisciplinary care teams organised around GP practices and workforce development;<br>Better prevention through risk identification, and self-management activities such as personal health budgets and telehealth.	Year of Care financial model and risk stratification;<br>Multidisciplinary care team meetings and neighbourhood care teams, integrated working within A&E departments, community integrated care centres;<br>Advanced Assistive technology partnership, joint working between paramedics and social care practitioners to respond quickly to 999 calls.	Aim

Keeble E, *et al. BMJ Open* 2019; 9:e026509. doi: 10.1136/bmjopen-2018-026509

Leeds	Whole population	Integrated commissioning with shared vision, outcomes and budget;<br>Integrated care through health and social care teams focused around GP practice populations and workforce development;<br>Prevention through self-management activities such as telehealth.	Risk stratification and year of care model;<br>Divestment from emergency department; funding where appropriate to reinvest into community-based services;<br>Fully integrated health and social care bed unit	Outcome
North West London	Whole population	Integrated commissioning with a shared vision, responsibility, budget allocation and information sharing;<br>Integrated care through joined up services, incentives, multidisciplinary care teams and care plans;<br>Prevention and self-management through personalisation.	Integrated care organisation;<br>Bespoke IT tool to access patient data for both patients and care providers to help prevent readmission.	Aim
Southend	Whole population with focus on high service users	Integrated commissioning through a Pioneer Strategic Group, with a shared budget and information management;<br>Integrated care through increased involvement of the voluntary sector and patients in co-design and workforce development.	7-day multidisciplinary teams;<br>Extension of the Single Point of Referral (SPOR) to reduce avoidable admissions and delayed transfers of care;<br>Hospital discharge-step down scheme.	Outcome
South Devon and Torbay	Whole population	Integrated commissioning and governance arrangements with shared objectives, information sharing and bringing together leaders in the health and social care system;	Support for a 24/7 hospice at home service through a chosen care home provider, with a team of specialist nurses and senior healthcare assistants, a rapid response service and night driver team;<br>Pilot of Sunday working over three consecutive weekends on 5 wards;	Outcome

Keeble E, *et al. BMJ Open* 2019; 9:e026509. doi: 10.1136/bmjopen-2018-026509

		Integrated care, not as an objective but an enabler, including an integrated care organisation providing acute, community and social care services 7 days a week, multidisciplinary teams including involvement of voluntary sector and workforce development;<br>Two programmes of work focused on better management of care and support for the elderly and young people.	Joined up IT including E-prescribing, E-booking and VitalPAC across healthcare organisations.	
South Tyneside	Whole population with focus on people who could benefit from initiatives on prevention, wellness promotion and self-care	Integrated commissioning and provision of care, through shared funding, joint decision-making, workforce development and information sharing.	Urgent care delivery group.<br>Predictive modelling to identify groups of patients vulnerable to hospital admission.	Aim
Stoke and North Staffordshire	Cancer and end-of-life care patients	Integrated service development through co-design by CCGs, Macmillan Cancer Support, Local Authorities and Public Health England to create accountable Service Integrator;<br>Integrated commissioning through shared arrangements across CCGs and social care and outcomes;<br>Integrated care through workforce development and co-design of care pathways.	Appointment of a principal provider for cancer and a principal provider for end of life care accountable for the determination of patient pathways through care.	-
Waltham Forest, East London and City	People at risk of hospital admission	Integrated commissioning through data sharing, joint	Whole System Demonstrator pilot.<br>Waltham Forest case management and rapid response programme.	Outcome

		health/social care assessment, joint contracting and payment; Integrated care through workforce development, restructuring of secondary care providers, greater involvement of the voluntary sector, rapid response and discharge tools, acute discharge support.		
Worcestershire	Whole population with focus on older people and people with long term conditions	Integrated commissioning through a shared funding of the oversight board, a shared vision, and community engagement.<br>Integrated care through service and clinical integration over organisational integration, reorganised acute care, multidisciplinary teams with involvement of the voluntary sector.<br>6 Transformation programme areas: Urgent Care; Out of hospital care; Specialised Commissioning; Acute Hospital Services; Future Lives; Children and Young People's Plan.	Virtual ward and integrated team projects.<br>Assistive technology for self-care and self-monitoring.<br>Improving Patient Flow strategy.<br>Personal Health Budgets.	Outcome

Keeble E, *et al. BMJ Open* 2019; 9:e026509. doi: 10.1136/bmjopen-2018-026509

2. Local Authority to Pioneer Lookup

Local Authority Code	Local Authority	Pioneer
E08000016	Barnsley	Barnsley
E06000050	Cheshire West and Chester	Cheshire
E06000049	Cheshire East	Cheshire
E06000052	Cornwall	Cornwall and Isles of Scilly
E06000053	Isles of Scilly	Cornwall and Isles of Scilly
E09000011	Greenwich	Greenwich
E09000019	Islington	Islington
E07000105	Ashford	Kent
E07000106	Canterbury	Kent
E07000107	Dartford	Kent
E07000108	Dover	Kent
E07000109	Gravesham	Kent
E07000110	Maidstone	Kent
E07000111	Sevenoaks	Kent
E07000112	Shepway	Kent
E07000113	Swale	Kent
E07000114	Thanet	Kent
E07000115	Tonbridge and Malling	Kent
E07000116	Tunbridge Wells	Kent
E08000035	Leeds	Leeds
E09000005	Brent	North West London
E09000009	Ealing	North West London
E09000013	Hammersmith and Fulham	North West London
E09000015	Harrow	North West London
E09000017	Hillingdon	North West London
E09000018	Hounslow	North West London
E09000020	Kensington and Chelsea	North West London
E09000033	Westminster	North West London
E06000027	Torbay	South Devon and Torbay
E07000044	South Hams	South Devon and Torbay
E07000045	Teignbridge	South Devon and Torbay
E08000023	South Tyneside	South Tyneside
E06000033	Southend-on-Sea	Southend
E07000192	Cannock Chase	Stoke and North Staffordshire
E07000194	Lichfield	Stoke and North Staffordshire
E07000195	Newcastle-under-Lyme	Stoke and North Staffordshire
E07000197	Stafford	Stoke and North Staffordshire
E07000198	Staffordshire Moorlands	Stoke and North Staffordshire
E06000021	Stoke-on-Trent	Stoke and North Staffordshire
E07000196	South Staffordshire	Stoke and North Staffordshire
E09000031	Waltham Forest	Waltham Forest and East London and City
E09000025	Newham	Waltham Forest and East London and City
E09000030	Tower Hamlets	Waltham Forest and East London and City
E07000234	Bromsgrove	Worcestershire

Keeble E, *et al. BMJ Open* 2019; 9:e026509. doi: 10.1136/bmjopen-2018-026509

E07000235	Malvern Hills	Worcestershire
E07000236	Redditch	Worcestershire
E07000237	Worcester	Worcestershire
E07000238	Wychavon	Worcestershire
E07000239	Wyre Forest	Worcestershire

Keeble E, *et al. BMJ Open* 2019; 9:e026509. doi: 10.1136/bmjopen-2018-026509

3. Emergency admission rates for individual local authorities within Pioneers at baseline and follow up, with percentage differences compared to baseline

Local Authority Code	Local Authority	Emergency Admission Rate			Percentage Difference	
		**Baseline**	**2014/15**	**2015/16**	**2014/15**	**2015/16**
E08000016	Barnsley	10,992	11,769	12,325	7.07%	12.13%
E06000049	Cheshire East	11,485	12,537	12,954	9.17%	12.80%
E06000050	Cheshire West and Chester	11,033	11,782	11,963	6.79%	8.42%
E06000052/53	Cornwall and Isles of Scilly	8,170	8,061	8,193	-1.33%	0.29%
E09000011	Greenwich	8,168	8,226	9,513	0.71%	16.47%
E09000019	Islington	6,324	6,320	6,096	-0.06%	-3.60%
E07000105	Ashford	8,708	9,785	10,506	12.37%	20.65%
E07000106	Canterbury	9,663	10,503	10,499	8.70%	8.66%
E07000107	Dartford	10,325	12,037	11,063	16.59%	7.15%
E07000108	Dover	9,201	10,411	10,809	13.14%	17.47%
E07000109	Gravesham	9,529	10,825	10,379	13.60%	8.92%
E07000110	Maidstone	9,553	10,470	10,219	9.60%	6.97%
E07000111	Sevenoaks	8,420	8,842	8,114	5.01%	-3.64%
E07000112	Shepway	8,921	9,566	10,274	7.24%	15.17%
E07000113	Swale	10,590	10,746	12,117	1.47%	14.42%
E07000114	Thanet	9,245	9,952	10,194	7.65%	10.27%
E07000115	Tonbridge and Malling	8,842	8,632	8,448	-2.37%	-4.46%
E07000116	Tunbridge Wells	9,188	8,631	7,483	-6.06%	-18.55%
E08000035	Leeds	11,399	9,605	10,155	-15.74%	-10.91%
E09000005	Brent	8,882	8,541	8,425	-3.84%	-5.15%
E09000009	Ealing	10,246	9,828	10,398	-4.08%	1.49%
E09000013	Hammersmith and Fulham	10,312	9,588	9,876	-7.03%	-4.23%
E09000015	Harrow	8,397	8,430	8,084	0.39%	-3.73%
E09000017	Hillingdon	9,753	9,636	9,346	-1.20%	-4.18%
E09000018	Hounslow	8,998	9,751	11,053	8.36%	22.83%
E09000020	Kensington and Chelsea	7,688	7,094	7,048	-7.73%	-8.32%
E09000033	Westminster	7,095	6,454	6,266	-9.04%	-11.70%
E07000044	South Hams	6,591	6,413	6,962	-2.69%	5.63%
E07000045	Teignbridge	7,657	8,182	9,275	6.86%	21.13%
E06000027	Torbay	7,998	8,296	10,173	3.72%	27.19%
E08000023	South Tyneside	11,153	10,445	11,150	-6.35%	-0.03%
E06000033	Southend-on-Sea	9,243	10,397	10,224	12.49%	10.61%
E07000192	Cannock Chase	10,438	10,611	10,083	1.66%	-3.40%
E07000194	Lichfield	8,754	9,424	9,971	7.65%	13.90%
E07000195	Newcastle-under-Lyme	11,919	12,034	12,625	0.96%	5.92%
E07000196	South Staffordshire	8,353	8,907	9,575	6.64%	14.63%
E07000197	Stafford	10,289	10,441	9,900	1.47%	-3.78%
E07000198	Staffordshire Moorlands	8,448	8,649	9,162	2.37%	8.45%
E06000021	Stoke-on-Trent	11,438	11,704	12,959	2.32%	13.29%

Keeble E, *et al. BMJ Open* 2019; 9:e026509. doi: 10.1136/bmjopen-2018-026509

E09000025	Newham	7,897	7,535	7,802	-4.58%	-1.20%
E09000030	Tower Hamlets	10,166	9,103	8,795	-10.45%	-13.49%
E09000031	Waltham Forest	9,489	9,333	8,242	-1.64%	-13.14%
E07000234	Bromsgrove	8,452	9,292	9,165	9.94%	8.43%
E07000235	Malvern Hills	8,289	7,937	7,360	-4.25%	-11.21%
E07000236	Redditch	10,556	10,650	11,534	0.89%	9.26%
E07000237	Worcester	9,858	9,180	9,705	-6.88%	-1.56%
E07000238	Wychavon	9,094	8,475	8,760	-6.81%	-3.68%
E07000239	Wyre Forest	7,855	7,366	7,511	-6.23%	-4.38%

4. Sensitivity analysis: excluding Stoke and North Staffordshire Pioneer

	**2014/15**	**2015/16**
**Model Coefficients** (p value)		
Intercept	-5.4249 (<0.0001)	-5.4267 (<0.0001)
Non-Pioneer/Pioneer	-0.0223 (0.2664)	-0.0223 (0.2673)
Baseline/Follow-Up	0.0487 (<0.0001)	0.0631 (<0.0001)
Interaction	-0.0308 (0.0464)	-0.0259 (0.1665)
**Percentage Difference** [95% confidence interval]		
Non-Pioneer	4.99 [3.81,6.18]	6.51 [5.18,7.86]
Pioneer	1.80 [-0.93,4.61]	3.78 [0.33,7.35]

Parallel trends interaction term p value = 0.3030

5. Sensitivity analysis: individual baseline years compared to 2014/15

**Baseline Year**	**2010/11**	**2011/12**	**2012/13**
**Parallel Trends P Value**	0.8608	0.3326	0.9899
**Model Coefficients** (p value)			
Intercept	-5.4148 (<0.0001)	-5.4504 (<0.0001)	-5.3476 (<0.0001)
Non-Pioneer/Pioneer	-0.0040 (0.8524)	-0.0051 (0.8016)	-0.0077 (0.6739)
Baseline/Time 2	0.0844 (<0.0001)	0.0512 (<0.0001)	0.0696 (<0.0001)
Interaction	-0.0298 (0.0860)	-0.0289 (0.0720)	-0.0260 (0.0189)
**Percentage Difference** [95% confidence interval]			
Non-Pioneer	8.81 [6.57,11.09]	5.26 [3.62,6.91]	7.21 [6.05,8.38]
Pioneer	5.61 [1.77,9.59]	2.26 [-0.70,5.30]	4.46 [2.45,6.51]

Keeble E, *et al. BMJ Open* 2019; 9:e026509. doi: 10.1136/bmjopen-2018-026509
