# Supplementary material for: Area level impacts on emergency hospital admissions of the integrated care and support pioneer programme in England: difference-in-differences analysis
Source: BMJ Open. 2019 Aug 18;9(8):e026509. doi: 10.1136/bmjopen-2018-026509 (PMC6701574; doi:10.1136/bmjopen-2018-026509)
Supplement: Author's manuscript [file bmjopen-2018-026509.draft_revisions.pdf]

BMJ Open

BMJ Open is committed to open peer review. As part of this commitment we make the peer review history of every article we publish publicly available.

When an article is published we post the peer reviewers' comments and the authors' responses online. We also post the versions of the paper that were used during peer review. These are the versions that the peer review comments apply to.

The versions of the paper that follow are the versions that were submitted during the peer review process. They are not the versions of record or the final published versions. They should not be cited or distributed as the published version of this manuscript.

BMJ Open is an open access journal and the full, final, typeset and author-corrected version of record of the manuscript is available on our site with no access controls, subscription charges or pay-per-view fees (http://bmjopen.bmj.com).

If you have any questions on BMJ Open's open peer review process please email info.bmjopen@bmj.com

BMJ Open

Area level impacts on emergency hospital admissions of the Integrated Care and Support Pioneer Programme in England: difference-in-differences analysis

Journal:	*BMJ Open*
Manuscript ID	bmjopen-2018-026509
Article Type:	Research
Date Submitted by the Author:	05-Sep-2018
Complete List of Authors:	Keeble, Eilís; The Nuffield Trust<br>Bardsley, M; The Health Foundation<br>Durand, Mary Alison; London School of Hygiene and Tropical Medicine, Policy Innovation Research Unit, Department of Health Services Research and Policy<br>Hoomans, Ties ; London School of Hygiene and Tropical Medicine, Policy Innovation Research Unit, Department of Health Services Research and Policy<br>Mays, Nicholas; London School of Hygiene and Tropical Medicine, Policy Innovation Research Unit, Department of Health Services Research and Policy
Keywords:	Quality in health care < HEALTH SERVICES ADMINISTRATION & MANAGEMENT, STATISTICS & RESEARCH METHODS, Organisation of health services < HEALTH SERVICES ADMINISTRATION & MANAGEMENT, Health policy < HEALTH SERVICES ADMINISTRATION & MANAGEMENT

SCHOLARONE™
Manuscripts

Area level impacts on emergency hospital admissions of the Integrated Care and Support Pioneer Programme in England: difference-in-differences analysis

Corresponding author: Eilís Keeble, The Nuffield Trust, 59 New Cavendish Street, London, W1G 7LP, United Kingdom, email: eilis.keeble@nuffieldtrust.org.uk, phone: 0207 631 8450

Martin Bardsley, The Health Foundation (Formerly of The Nuffield Trust), London, United

Mary Alison Durand, Policy Innovation Research Unit, Department of Health Services Research and Policy, London School of Hygiene and Tropical Medicine, London, United Kingdom

Ties Hoomans, Policy Innovation Research Unit, Department of Health Services Research and Policy, London School of Hygiene and Tropical Medicine, London, United Kingdom

Nicholas Mays, Policy Innovation Research Unit, Department of Health Services Research and Policy, London School of Hygiene and Tropical Medicine, London, United Kingdom

Word Count: 2,858

For peer review only

ABSTRACT

Objective

To examine whether any differential change in emergency admissions could be attributed to Pioneer status by comparing Pioneer and non-Pioneer populations from a pre-Pioneer baseline period (2010/11 to 2012/13) to two follow-up periods: 2014/15 and 2015/16.

Design

Difference-in-differences analysis of emergency hospital admissions from Hospital Episode Statistics (HES).

Setting

Local authorities in England classified as either Pioneer or non-Pioneer.

Participants

Emergency admissions to all NHS hospitals in England with local authority determined by area of residence of the patient.

Intervention

Wave 1 of the Integrated Care and Support Pioneer Programme announced in November 2013.

Primary Outcome Measure

Hospital emergency admissions.

Results

The increase in the Pioneer emergency admission rate from baseline to 2014/15 was smaller at 1.42 per cent and significantly different from that of the non-Pioneers at 4.44 per cent (p=0.0161). The increase in the Pioneer emergency admission rate from baseline to 2015/16 was again smaller than for the non-Pioneers but the difference was not statistically significant (p=0.1272).

Conclusions

It is ambitious to expect unequivocal changes in a high level and indirect indicator of care integration such as emergency hospital admissions to arise as a result of the changes in local health and social care provision across organisations brought about by the Pioneers in their

For peer review only

early years and we should treat any indication that the Pioneers have had such an impact with caution. Nevertheless, there does seem to be an indication from the current analysis that there were some changes in hospital use associated with the first year of Pioneer status that are worthy of further exploration.

STRENGTHS AND LIMITATIONS OF THIS STUDY

1. This study adds to the evidence of the impact of system-wide approaches to integrating health and social care, like the Pioneer Programme, using advanced statistical methods to analyse HES and determine whether the Pioneers reduced emergency admissions.
2. Emergency admissions data are continuously collected which makes them more susceptible to change than many other measures of health and social care integration and reducing them is often cited as a key goal of new models of care.
3. Analysing the Pioneer sites collectively ensured they covered a diverse range of areas and were unlikely to be systematically different at baseline from the non-Pioneers.
4. It is difficult to find a true counter-factual population to compare with the Pioneers as many other initiatives around health and social care integration were developed in other areas of the country almost simultaneously.
5. The Pioneers invested in a collection of health and social care integration strategies and interventions; detecting the causes and effects of these specific initiatives would require local datasets and limit the generalisability of findings to the Pioneers as a whole.

KEYWORDS

Organisation of health services, quality in health care, health policy, statistics and research methods

For peer review only

INTRODUCTION

In November 2013, the Integrated Care and Support Pioneer programme was initiated in England. The prime stated objective was to meet people's needs better and improve service users' experience of care by encouraging more integrated ways of working between local NHS and social care providers.[1]

In the first wave of the programme the UK government selected 14 Pioneer areas from a round of competitive applications, having being identified as the "most ambitious and visionary" in their plans for health and social care integration.[2] Each Pioneer was given access to support and expertise over a five-year period and a one-off fund of £90,000 to help with initial development. A second wave of 11 Pioneer areas was subsequently announced in January 2015. These are excluded from the present analysis as there are insufficient follow up time points available currently for an interpretable trend analysis.

The specific interventions to be implemented were not prescribed but the Pioneers broadly shared the same vision for the future of the health and social care system by seeking to create a 'whole system' of integrated care involving all local bodies and professional groups organised around the needs of individuals and their informal carers.[3]

Many of the Pioneers planned to focus on older people, people with multiple long term conditions, people at high risk of hospitalisation and families with complex care needs. A number of Pioneers aimed to reduce reliance on emergency hospital care by introducing preventive strategies to avoid the need for acute hospitalisation. Such strategies offered the hope of better quality care and experiences for patients, and potentially the better use of limited resources by reducing the costs of what was perceived to be more expensive hospital care. The focus on reducing emergency use of hospital care was given greater emphasis in the Pioneers' plans as financial austerity bit more deeply into local health care budgets after 2013.[4]

As a consequence of the focus on emergency hospital care, the success of integrated care initiatives has often been presented, at least in part, in terms of the ability to reduce the need for emergency hospital admissions and to reduce emergency admission rates.[5] Reducing emergency admission rates has been a feature of English health policy over the past decade

For peer review only

and continues to be one of the most commonly used measures of success.[6–8] To date, however, there has been little evidence of initiatives successfully reducing emergency admissions.[9–11]

In this paper, we investigate whether emergency admissions among the first wave Pioneers diverged from rates in other parts of England using a difference-in-differences approach. Though the causality may be unclear in terms of which elements of the programme, if any, led to any differential change observed, such an analysis can be justified as a necessary step in understanding the impacts of a major initiative such as the integrated care Pioneer programme. The underlying hypothesis is that the cumulative effect of the specific initiatives embedded in each Pioneer programme would bring about sufficient change in emergency hospital care use that it would be detectable at the level of the whole population of the Pioneers. The analysis is part of a wider programme of evaluation of the Pioneers (http://piru.lshtm.ac.uk/projects/current-projects/integrated-care-pioneers-evaluation.html).

METHODS

To examine whether differential change in emergency admissions could be attributed to Pioneer status a difference-in-differences approach was used. This compared time series data on NHS hospital emergency admission rates between Pioneer and non-Pioneer local authority populations from a baseline period of 2010/11-2012/13 to two follow-up periods: 2014/15 and 2015/16. Difference-in-differences measures the effect of the intervention (the Pioneer programme) by looking at the change in emergency admissions in the two groups and quantifies whether or not the population within the Pioneer programme experiences a change that is significantly different to the comparison group, the non-Pioneers.

Defining Pioneer Areas

The Pioneer areas were not based on a consistent set of formal geographies and, in the absence of data on those who directly benefited from the Pioneers' planned interventions, an exercise was undertaken to map each Pioneer to the local authorities it covered (see online supplementary material appendices for lookup table). Many of the Pioneers were based on clinical commissioning group (CCG) boundaries instead of local authority. In these cases all the local authorities the CCG overlapped with were included as part of the Pioneer's

For peer review only

geographical area. Local authority boundaries were chosen instead of CCG because the time series of population estimates available for local authorities is longer.

The local authorities which were linked to the second wave of Pioneers, initiated in January 2015, were excluded from all analyses and not included in either the Pioneer or non-Pioneer populations.

Data Sources and Preparation

The analysis used inpatient Hospital Episode Statistics (HES) collated by NHS Digital.[12] HES is a pseudonymous patient level dataset that records basic features of admissions to hospital including a field that indicates an emergency admission (admission methods starting with "2"). The numbers of emergency admissions for individual years 2007/08 to 2015/16 were calculated by summing the number of completed spells in hospital (period from admission to discharge) by local authority of residence, age group (0-19, 20-39, 40-59, 60-79, 80+) and sex. Emergency admission rates were calculated as the total number of emergency admissions divided by the mid-year population estimate for each local authority obtained from the Office for National Statistics (ONS).[13] For the rates, the emergency admissions were directly standardised using the age and sex structure of the 2015 mid-year population estimates for England. A baseline of the average of the emergency admissions in the financial years 2010/11 to 2012/13 was created as the period before the initiation of the Pioneer programme in late 2013. Local authority-level deprivation decile was taken from the Index of Multiple Deprivation 2015.[14]

Statistical Analysis

To get an understanding of the similarity between the Pioneer and non-Pioneer populations, baseline emergency admissions and demographics of the two populations were examined. The emergency admission rates for the Pioneers and non-Pioneers were compared graphically over the period prior to the initiation of the programme (from 2007/08 to 2012/13) to assess whether the trends were parallel and suitable for difference-in-differences analysis.

A crude difference in difference comparison was performed by looking at the change in the emergency admission rate for the Pioneers and non-Pioneers. Percentage differences

For peer review only

between the baseline period and the two follow up points of 2014/15 and 2015/16 were calculated.

To determine whether the change in emergency admissions in the Pioneers was significantly different from the change in the non-Pioneers, we performed difference-in-differences regression analysis, using a negative binomial regression model. We deemed a Poisson model for count data not suitable because the emergency admission data were over-dispersed. A count of emergency admissions in each local authority was used as the dependent variable. To model the emergency admissions counts as a rate, an offset of the log of the population size in each local authority was incorporated into the model. The independent variables were Pioneer status (Pioneer/non-Pioneer), time (baseline/follow-up time point of either 2014/15 or 2015/16) and an interaction term of Pioneer status*time. The interaction term provides the model coefficient to differentiate whether or not the difference in the differences in emergency admissions between the two groups is statistically significant. The model accounted for the repeated measures from each local authority and was adjusted for population age group, sex and area-level deprivation.

SAS 9.4® was used for all analyses.

Patient and Public Involvement

Patients were not involved in the design or interpretation of this particular analysis since it is entirely reliant on routine data. However, patient and public representatives are involved in the wider evaluation of which this analysis forms a part and were involved in the selection and peer review of the initial proposal on which this analysis is based.

RESULTS

Baseline Characteristics

The characteristics of the Pioneers and non-Pioneers during the baseline period of 2010/11 to 2012/13 are summarised in Table 1. The Pioneers were made up of 49 local authorities, which encompassed 17 percent of the English population in 2015, with a higher average local authority population size than non-Pioneers.[13] The proportions of the population aged 65 and over, or female, were similar between the two groups; area-level deprivation in the

For peer review only

Pioneers was slightly higher. At baseline, the Pioneers had a higher emergency hospital admission rate than the non-Pioneers.

Table 1 Baseline Characteristics of the Pioneer and non-Pioneer populations

**Characteristic**	**Pioneers**	**Non-Pioneers**
Number of Local Authorities	49	244
Number of Pioneer Areas	14	-
Total Population	9,083,051	37,137,613
Population Per Local Authority	*185,368*	*152,203*
Proportion Population Under 20	24%	24%
Proportion Population Aged 65+	16%	17%
Proportion Population Female	50%	50%
Average IMD Score	22.8	20.7
Total Emergency Admissions	878,855	3,578,799
Emergency Admissions Per Local Authority	*17,934*	*14,667*
Crude Emergency Admission Rate Per 100,000 Population	9,676	9,637
Directly Standardised Emergency Admission Rate Per 100,000 Population	10,013	9,705

Trend Analysis

Figure 1 shows the emergency admission rates for the Pioneers and non-Pioneers Between 2007/08 and 2012/13. The trends seem close to parallel, with Pioneers consistently having a higher emergency admission rate, indicating limited trends bias in our difference-in-differences analysis.

Crude Change

The change in the unadjusted emergency admission rate between the baseline period and 2014/15 was less for the Pioneers than the non-Pioneers (see Table 2). On average, the Pioneers had a decrease of 0.22 per cent in the emergency admission rate between baseline and 2014/15 compared to an increase of 3.43 per cent for the non-Pioneers, with difference-in-differences of 3.65%. The estimated effects of the Pioneers was not as large over a longer time period, with difference-in-differences at 3.30% in 2015/16.

Table 2 Emergency admission rates for Pioneers and Non-Pioneers at baseline and follow up, with percentage differences compared to baseline and difference in difference between non Pioneer and Pioneer

	Emergency Admission Rate (per 100,000 population)			Percentage Difference		Difference-in-Differences*	
	Baseline	2014/15	2015/16	2014/15	2015/16	2014/15	2015/16
Non-Pioneer	9,705	10,038	10,402	3.43%	7.18%	3.65	3.30

For peer review only

Pioneer	10,013	9,991	10,402	-0.22%	3.88%		

** Difference between the non-Pioneer and Pioneer percentage difference, positive value indicates non-Pioneer change is greater*

It is important to note that trends for the individual Pioneers varied. For example, eight of the fourteen Pioneers had an increase in the emergency admission rate between baseline and 2014/15, while the percentage difference for the Pioneers as a whole was a slight decrease (see Table 3). There was also variation within Pioneers (see online supplementary material appendices for table); for example, the constituent local authorities comprising the Waltham Forest, East London and City Pioneer had declines in emergency admission rates ranging from -12.5% (Tower Hamlets) to -0.4% (Newham) between baseline and 2014/15.

Table 3 Emergency admission rates for individual pioneers at baseline and follow up, with percentage differences compared to baseline

Pioneer (Number of LAs)	Emergency Admission Rate (per 100,000 population)			Percentage Difference	
	Baseline†	2014/15	2015/16	2014/15	2015/16
Barnsley (1)	11,873	12,942	13,667	9.01%	15.12%
Cheshire (2)	10,160	10,912	11,303	7.40%	11.24%
Cornwall and Isles of Scilly (2)	9,001	8,747	9,081	-2.83%	0.89%
Greenwich (1)	9,536	9,661	11,382	1.31%	19.36%
Islington (1)	11,397	11,607	11,219	1.85%	-1.56%
Kent (12)	9,204	9,797	10,017	6.44%	8.83%
Leeds (1)	11,900	9,848	10,728	-17.24%	-9.84%
North West London (8)	10,306	10,125	10,416	-1.76%	1.07%
South Devon and Torbay (3)	8,668	9,013	10,597	3.98%	22.26%
South Tyneside (1)	12,670	11,873	13,325	-6.29%	5.17%
Southend (1)	9,506	10,735	10,797	12.93%	13.58%
Stoke and North Staffordshire (7)	10,856	11,118	11,830	2.42%	8.97%
Waltham Forest, East London and City (3)	12,367	11,684	11,041	-5.52%	-10.72%
Worcestershire (6)	8,633	8,429	8,919	-2.37%	3.31%

†Averaged emergency admission rate for 2010/11 – 2012/13

Difference-in-Differences Regression

After adjusting for age, sex and area-level deprivation, our difference-in-differences regression analysis found the change in emergency admission rates for the Pioneers between baseline and 2014/15 was smaller and significantly different from that of the non-Pioneers (p=0.0161) (see Table 4). The Pioneer emergency admission rate increased by 1.42 per cent

For peer review only

compared to 4.44 per cent in the non-Pioneers. When comparing baseline and 2015/16, the analysis still indicated that the change in emergency admissions for the Pioneers was smaller at 4.97 per cent compared to 7.65 percent for the non-Pioneers but the difference was not statistically significant (p=0.1272) (see online supplementary material appendices for full model results).

Table 4 Difference in difference model coefficients and calculated percentage difference in emergency admissions for Pioneers and Non-Pioneers, adjusted for age, sex and area-level deprivation

	**2014/15**	**2015/16**
**Model Coefficients** (p value)		
Intercept	-2.4597 (<0.0001)	-2.5102 (<0.0001)
Non-Pioneer/Pioneer	-0.0038 (0.8352)	-0.0035 (0.8466)
Baseline/Time 2	0.0435 (<0.0001)	0.0737 (<0.0001)
Interaction	-0.0293 (0.0161)	-0.0252 (0.1272)
**Percentage Difference** [95% confidence interval]		
Non-Pioneer	4.44 [3.43,5.47]	7.65 [6.49,8.83]
Pioneer	1.42 [-0.77,3.66]	4.97 [1.81,8.23]

DISCUSSION

The Integrated Care Pioneers represent one important example of how English health and care services have been exploring new ways of working across organisational boundaries. The aims of the individual Pioneers varied,[4] but most had a common interest in providing care and support that was intended to reduce the need for urgent care services and lead to a reduction in emergency hospital admissions. After comparing changes in emergency admissions from a 3-year pre-Pioneer baseline period between Pioneer populations and non-Pioneer populations, we found that, despite higher emergency admission rates in the baseline period, the increase in emergency admission rates was lower for the Pioneers than the non-Pioneers. This lower increase was statistically significant for the comparison between baseline and 2014/15 (p=0.0161) but not for the comparison between baseline and 2015/16 (p=0.1272).

This type of population level analysis can help provide some independent evidence of the likely scale of changes within an area associated with integrated care initiatives and curb some of the more zealous rhetoric for or against integrated health and social care and related

For peer review only

changes in service delivery. Looking at emergency admission data on this scale means the outcome of interest is based on a relatively large number of events and the data are collected continuously – making them useful as a measure of potential programme impact. This is in contrast to a range of other potential measures of health and social care integration at community level that are less sensitive to short term change such as annual patient experience surveys. The size and range of geographical areas covered by both the Pioneers and non-Pioneers should mean that differences in factors such as supply of social care services or acute hospital beds and the process of collecting data are unlikely to be systematically different between the two groups beyond any changes associated with Pioneer status.

In addition to the introduction of the Pioneer programme, there have been subsequent parallel changes in the wider policy context both in terms of specific health policies such as the Better Care Fund,[15] the overall level of funding for both health and social care in a period of unprecedented financial austerity and,[16] from 2014 onwards, the New Care Model Vanguards, arising from the strategic directions set out in the Five Year Forward View.[17] This means that the ideas behind integration that prompted the Pioneers and the types of interventions that have been developed are no longer (if they ever were) unique to these areas and are being implemented across the country. Therefore, a true counter-factual population is difficult to find. This may in part explain why the difference between the Pioneers and non-Pioneers reduced between baseline and 2015/16 compared with baseline and 2014/15 as the behaviour of the non-Pioneers becomes increasingly similar to the Pioneers.[18]

A more detailed understanding of the impacts of the Pioneers would be gained by a more targeted analytical approach using information on the specific initiatives implemented in each Pioneer and data on the exact populations in receipt of these initiatives (this is being attempted in another component of the Pioneer evaluation). While this might yield gains in terms of causal inference in that changes could potentially be attributed to a specific set of local actions, such an analysis might lose the ability to describe the totality of change across a system and an entire population. This is important to note as the Pioneers were intended to be a complex mix of specific service changes and initiatives, supported by a wider pattern of infrastructural changes at the level of the local health and social care system. As Erens and colleagues noted *"What it meant to be 'a Pioneer' varied between sites and between*

For peer review only

individuals within sites. At various times it was apparent that Pioneer status meant one or more of the following:

• a 'badge' for a locality signifying national recognition of innovation and progress in integrating care
• an enabler of the existing local plan for transformation
• a particular governance arrangement, for example a Board that brought all system leaders and their organisations around the table
• a collection of discrete workstreams, characteristically covering a combination of different groups of users and infrastructure projects (for example, information sharing, workforce development, etc.)
• a specific new integrated service, such as a frailty service
• an ethos or way of thinking about and providing care, rather than a specific plan or set of initiatives"[4]

Other studies have looked at schemes with an aspiration to reduce the need for urgent hospital care through better coordinated health and care services, and with an emphasis on preventing admissions. Success is typically assessed in terms of reduction in emergency hospital admissions and various previous evaluations show that this has been difficult to achieve.[9–11] Despite the intense policy interest in how different forms of service delivery can reduce emergency admissions, there are few, if any, unequivocal success stories. Against this backdrop, the modest changes observed across the 14 wave one Pioneer areas in their first two years look promising. However, when exploring the extent to which the observed changes are likely to be related causally to Pioneer status, it should be noted that:

a. the effect appears to be temporary: and as such the effect may have been linked to changes that took place in the early stages of the Pioneers but were not sustained; or the non-Pioneer areas introduced changes which have subsequently reduced the difference between them and the Pioneers; and
b. the changes in emergency admissions were not shown in all places and even varied between local authority areas within the same Pioneer.

For peer review only

Conclusion

It is ambitious to expect unequivocal changes in a single high level and indirect indicator of care integration such as emergency hospital admissions to arise as a result of changes in local health and care provision across organisations brought about by the Pioneers in their early years and we should treat any claims that the Pioneers have had such an impact with caution. Nevertheless, there does seem to be some evidence from the current analysis that there were some changes in hospital use associated with the first year of Pioneer status that are worthy of further exploration. At the very least, this analysis shows that Pioneer status does not seem to have been associated with a relative deterioration in performance in terms of emergency hospital use.

FIGURE LEGEND

Figure 1 Emergency admissions for Pioneers and Non-Pioneers, directly standardised rates per 100,000 standard population (baseline years as shaded area)

AUTHOR CONTRIBUTIONS

EK undertook the analysis and with MB drafted the initial paper. MB and NM contributed to design of the study. EK, MB, MA, TH and NM contributed to interpretation of findings and revisions of the paper.

ACKNOWLEDGEMENTS

This work uses data provided by patients and collected by the NHS as part of their care and support. Read more at https://www.nuffieldtrust.org.uk/about/corporate-policies#information-security-and-data.

Hospital Episode Statistics data (year range 2007/08–2015/16) Copyright © (2018), NHS Digital. Re-used with the permission of NHS Digital. All rights reserved.

FUNDING

This work was supported by the NIHR Policy Research Programme, *Evaluation of the Integrated Care and Support Pioneers Programme in the context of new funding*

arrangements for integrated care in England (2015-2020), PR-R10-0514-25001. The views expressed in the publication are those of the authors and are not necessarily those of the NHS, the NIHR, the Department of Health and Social Care, its arm's length bodies or other Government Departments.

For peer review only

REFERENCES

[1] NHS England. Integrated care pioneers announced. 2013. Available: https://www.england.nhs.uk/2013/11/interg-care-pioneers/. [Accessed: 01-Aug-2018].

[2] Department of Health. Letter inviting expressions of interest for health and social care integration "Pioneers". 2013. Available: https://www.gov.uk/government/publications/social-care-integration-pioneers. [Accessed: 01-Aug-2018].

[3] National Collaboration for Integrated Care and Support. Integrated Care and Support: Our Shared Commitment. 2013. Available: https://assets.publishing.service.gov.uk/government/uploads/system/uploads/attachment_data/file/198748/DEFINITIVE_FINAL_VERSION_Integrated_Care_and_Support_-_Our_Shared_Commitment_2013-05-13.pdf. [Accessed: 01-Aug-2018].

[4] Erens B, Wistow G, Mournier-Jack S, et al. Early evaluation of Integrated Care and Support Pioneers Programme: Final Report. Policy Innovation Research Unit. 2015. Available: http://piru.lshtm.ac.uk/assets/files/Early_evaluation_of_IC_Pioneers_Final_Report.pdf. [Accessed: 01-Aug-2018].

[5] Raleigh V, Bardsley M, Smith P, et al. Integrated care and support Pioneers: Indicators for measuring the quality of integrated care: Final report. Policy Innovation Research Unit. 2014. Available: http://piru.lshtm.ac.uk/assets/files/IC_and_support_Pioneers-Indicators.pdf. [Accessed: 01-Aug-2018].

[6] National Audit Office. Emergency admissions to hospital: managing the demand. National Audit Office. 2013. Available: https://www.nao.org.uk/wp-content/uploads/2013/10/10288-001-Emergency-admissions.pdf. [Accessed: 01-Aug-2018].

[7] Wallace E, Smith SM, Fahey T, et al. Reducing emergency admissions through community based interventions. *BMJ* 2016;352:h6817 doi:10.1136/bmj.h6817 [published Online first: 28 January 2016].

[8] Lloyd T, Wolters A, Steventon A. The impact of providing enhanced support for care home residents in Rushcliffe. The Health Foundation. 2017. Available: https://www.health.org.uk/publication/impact-enhanced-support-rushcliffe. [Accessed: 01-Aug-2018].

[9] Bardsley M, Steventon A, Smith J, et al. Evaluating integrated and community-based care: How do we know what works? The Nuffield Trust. 2013. Available: https://www.nuffieldtrust.org.uk/files/2017-01/evaluating-integrated-community-care-web-final.pdf. [Accessed: 01-Aug-2018].

[10] Roland M, Lewis R, Steventon A, et al. Case management for at-risk elderly patients in the English integrated care pilots: observational study of staff and patient experience and secondary care utilisation. *Int J Integr Care* 2012;12(5) http://doi.org/10.5334/ijic.850.

[11] Purdy S, Paranjothy S, Huntley A, et al. Interventions to reduce unplanned hospital admission: a series of systematic reviews. National Institute for Health Research. 2012. Available: http://www.bristol.ac.uk/media-library/sites/primaryhealthcare/migrated/documents/unplannedadmissions.pdf. [Accessed: 01-Aug-2018].

[12] NHS Digital. Hospital Episode Statistics data 2007/08-2015/16.

[13] Office for National Statistics. Population estimates 2007-2015. Available: https://www.ons.gov.uk/peoplepopulationandcommunity/populationandmigration/populationestimates. [Accessed: 14-May-2018].

[14] Ministry of Housing, Communities and Local Government. English indices of deprivation 2015. Available: https://www.gov.uk/government/statistics/english-indices-of-deprivation-2015. [Accessed: 12-Jun-2018].

[15] NHS England. Better Care Fund. Available: https://www.england.nhs.uk/ourwork/part-rel/transformation-fund/bcf-plan/. [Accessed: 14-May-2018].

[16] Robertson R, Wenzel L, Thompson J, et al. Understanding NHS financial pressures: How are they affecting patient care? The Kings Fund. 2017.

[17] NHS. NHS Five Year Forward View. 2014. Available: https://www.england.nhs.uk/wp-content/uploads/2014/10/5yfv-web.pdf. [Accessed: 01-Aug-2018].

[18] Chen YF, Hemming K, Stevens AJ, et al. Secular trends and evaluation of complex interventions: the rising tide phenomenon. *BMJ Qual Saf 2016;*25(5)303–310 doi: 10.1136/bmjqs-2015-004372 [published Online first: 06 Oct 2015].

For peer review only

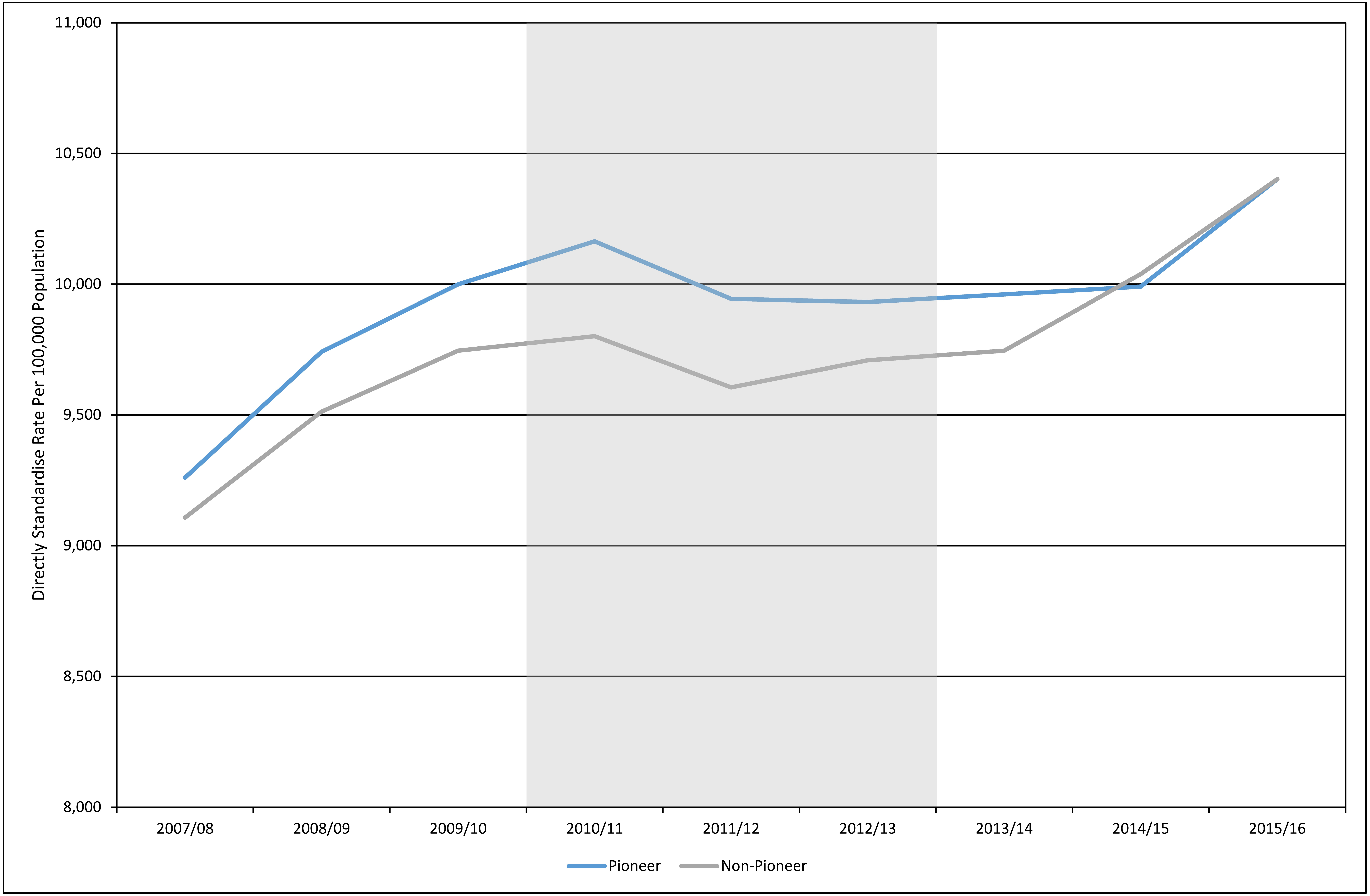
11,000
10,500
10,000
9,500
9,000
8,500
8,000
Directly Standardise Rate Per 100,000 Population
2007/08
2008/09
2009/10
2010/11
2011/12
2012/13
2013/14
2014/15
2015/16
Pioneer
Non-Pioneer

SUPPLEMENTARY MATERIAL

1. Local Authority to Pioneer Lookup
2. Emergency admission rates for individual local authorities within Pioneers
3. Difference-in-differences baseline and 2014/15 full model coefficients
4. Difference-in-differences baseline and 2015/16 full model coefficients

1. Local Authority to Pioneer Lookup

Local Authority Code	Local Authority	Pioneer
E08000016	Barnsley	Barnsley
E06000050	Cheshire West and Chester	Cheshire
E06000049	Cheshire East	Cheshire
E06000052	Cornwall	Cornwall and Isles of Scilly
E06000053	Isles of Scilly	Cornwall and Isles of Scilly
E09000011	Greenwich	Greenwich
E09000019	Islington	Islington
E07000105	Ashford	Kent
E07000106	Canterbury	Kent
E07000107	Dartford	Kent
E07000108	Dover	Kent
E07000109	Gravesham	Kent
E07000110	Maidstone	Kent
E07000111	Sevenoaks	Kent
E07000112	Shepway	Kent
E07000113	Swale	Kent
E07000114	Thanet	Kent
E07000115	Tonbridge and Malling	Kent
E07000116	Tunbridge Wells	Kent
E08000035	Leeds	Leeds
E09000005	Brent	North West London
E09000009	Ealing	North West London
E09000013	Hammersmith and Fulham	North West London
E09000015	Harrow	North West London
E09000017	Hillingdon	North West London
E09000018	Hounslow	North West London
E09000020	Kensington and Chelsea	North West London
E09000033	Westminster	North West London
E06000027	Torbay	South Devon and Torbay
E07000044	South Hams	South Devon and Torbay
E07000045	Teignbridge	South Devon and Torbay
E08000023	South Tyneside	South Tyneside
E06000033	Southend-on-Sea	Southend
E07000192	Cannock Chase	Stoke and North Staffordshire
E07000194	Lichfield	Stoke and North Staffordshire

For peer review only - http://bmjopen.bmj.com/site/about/guidelines.xhtml

E07000195	Newcastle-under-Lyme	Stoke and North Staffordshire
E07000197	Stafford	Stoke and North Staffordshire
E07000198	Staffordshire Moorlands	Stoke and North Staffordshire
E06000021	Stoke-on-Trent	Stoke and North Staffordshire
E07000196	South Staffordshire	Stoke and North Staffordshire
E09000031	Waltham Forest	Waltham Forest and East London and City
E09000025	Newham	Waltham Forest and East London and City
E09000030	Tower Hamlets	Waltham Forest and East London and City
E07000234	Bromsgrove	Worcestershire
E07000235	Malvern Hills	Worcestershire
E07000236	Redditch	Worcestershire
E07000237	Worcester	Worcestershire
E07000238	Wychavon	Worcestershire
E07000239	Wyre Forest	Worcestershire

2. Emergency admission rates for individual local authorities within Pioneers at baseline and follow up, with percentage differences compared to baseline

Local Authority Code	Local Authority Name	Emergency Admission Rate			Percentage Difference	
		Baseline	2014/15	2015/16	2014/15	2015/16
E08000016	Barnsley	11,873	12,942	13,667	9.0%	15.1%
E06000050	Cheshire West and Chester	10,315	11,047	11,403	7.1%	10.6%
E06000049	Cheshire East	10,028	10,806	11,231	7.8%	12.0%
E06000052	Cornwall (incl. Isles of Scilly)	9,001	8,747	9,081	-2.8%	0.9%
E09000011	Greenwich	9,536	9,661	11,382	1.3%	19.4%
E09000019	Islington	11,397	11,607	11,219	1.8%	-1.6%
E07000105	Ashford	8,598	9,584	10,556	11.5%	22.8%
E07000106	Canterbury	10,463	11,335	11,470	8.3%	9.6%
E07000107	Dartford	9,776	11,347	10,620	16.1%	8.6%
E07000108	Dover	9,152	10,411	10,924	13.8%	19.4%
E07000109	Gravesham	9,167	10,389	10,113	13.3%	10.3%
E07000110	Maidstone	8,447	9,181	9,112	8.7%	7.9%
E07000111	Sevenoaks	8,120	8,500	8,210	4.7%	1.1%
E07000112	Shepway	9,220	10,013	10,925	8.6%	18.5%
E07000113	Swale	9,341	9,333	10,697	-0.1%	14.5%
E07000114	Thanet	10,426	11,122	11,424	6.7%	9.6%
E07000115	Tonbridge and Malling	8,539	8,408	8,384	-1.5%	-1.8%
E07000116	Tunbridge Wells	8,919	7,983	7,730	-10.5%	-13.3%
E08000035	Leeds	11,900	9,848	10,728	-17.2%	-9.8%
E09000005	Brent	10,371	10,017	9,948	-3.4%	-4.1%
E09000009	Ealing	11,702	11,274	12,147	-3.7%	3.8%
E09000013	Hammersmith and Fulham	12,015	11,358	11,770	-5.5%	-2.0%
E09000015	Harrow	8,769	8,848	8,588	0.9%	-2.1%
E09000017	Hillingdon	10,906	10,886	10,660	-0.2%	-2.2%

For peer review only - http://bmjopen.bmj.com/site/about/guidelines.xhtml

E09000018	Hounslow	10,316	11,358	13,250	10.1%	28.4%
E09000020	Kensington and Chelsea	8,720	8,138	8,035	-6.7%	-7.9%
E09000033	Westminster	9,404	8,502	8,214	-9.6%	-12.7%
E06000027	Torbay	9,497	9,948	12,322	4.7%	29.7%
E07000044	South Hams	7,752	7,815	8,491	0.8%	9.5%
E07000045	Teignbridge	8,409	8,824	10,183	4.9%	21.1%
E08000023	South Tyneside	12,670	11,873	13,325	-6.3%	5.2%
E06000033	Southend-on-Sea	9,506	10,735	10,797	12.9%	13.6%
E07000192	Cannock Chase	10,485	10,847	10,298	3.4%	-1.8%
E07000194	Lichfield	8,845	9,610	10,172	8.7%	15.0%
E07000195	Newcastle-under-Lyme	11,457	11,591	12,479	1.2%	8.9%
E07000197	Stafford	10,510	10,586	10,324	0.7%	-1.8%
E07000198	Staffordshire Moorlands	9,120	9,288	9,956	1.8%	9.2%
E06000021	Stoke-on-Trent	13,447	13,586	15,386	1.0%	14.4%
E07000196	South Staffordshire	8,528	9,187	10,006	7.7%	17.3%
E09000031	Waltham Forest	12,123	11,577	10,288	-4.5%	-15.1%
E09000025	Newham	12,379	12,336	12,118	-0.4%	-2.1%
E09000030	Tower Hamlets	12,867	11,263	10,789	-12.5%	-16.1%
E07000234	Bromsgrove	8,063	8,625	9,200	7.0%	14.1%
E07000235	Malvern Hills	7,878	7,460	7,439	-5.3%	-5.6%
E07000236	Redditch	10,601	10,642	11,822	0.4%	11.5%
E07000237	Worcester	9,417	8,768	9,459	-6.9%	0.4%
E07000238	Wychavon	8,255	7,923	8,305	-4.0%	0.6%
E07000239	Wyre Forest	7,998	7,675	7,913	-4.0%	-1.1%

3. Difference-in-differences baseline and 2014/15 full model coefficients

Parameter	Estimate	Standard Error	95% Confidence Limits		Z	Pr > |Z|
Intercept						
	-2.4597	0.0276	-2.5138	-2.4056	-89.12	<.0001
Wave						
Non-Pioneer	ref	ref	ref	ref	ref	ref
Pioneer	-0.0038	0.0182	-0.0395	0.0319	-0.21	0.8352
Time						
	0.0435	0.0050	0.0337	0.0532	8.75	<.0001
Interaction Time*Wave						
	-0.0293	0.0122	-0.0533	-0.0054	-2.41	0.0161
Age Group						
00-19	ref	ref	ref	ref	ref	ref
20-39	-0.1317	0.0116	-0.1544	-0.1089	-11.35	<.0001
40-59	-0.0570	0.0131	-0.0826	-0.0313	-4.35	<.0001

For peer review only - http://bmjopen.bmj.com/site/about/guidelines.xhtml

60-79	0.7052	0.0150	0.6758	0.7346	47.01	<.0001
80+	1.8660	0.0155	1.8355	1.8964	120.09	<.0001
Sex						
Male	ref	ref	ref	ref	ref	ref
Female	-0.0030	0.0028	-0.0085	0.0025	-1.07	0.2830
Index of Multiple Deprivation Decile						
1	ref	ref	ref	ref	ref	ref
2	-0.1034	0.0338	-0.1696	-0.0372	-3.06	0.0022
3	-0.1234	0.0349	-0.1918	-0.0551	-3.54	0.0004
4	-0.1731	0.0329	-0.2376	-0.1086	-5.26	<.0001
5	-0.2294	0.0352	-0.2984	-0.1603	-6.51	<.0001
6	-0.2203	0.0319	-0.2829	-0.1577	-6.90	<.0001
7	-0.3115	0.0311	-0.3723	-0.2506	-10.03	<.0001
8	-0.3164	0.0323	-0.3798	-0.2531	-9.79	<.0001
9	-0.3794	0.0274	-0.4330	-0.3257	-13.87	<.0001
10	-0.4324	0.0290	-0.4892	-0.3756	-14.92	<.0001

4. Difference-in-differences baseline and 2015/16 full model coefficients

**Parameter**	**Estimate**	**Standard Error**	**95% Confidence Limits**		**Z**	**Pr > |Z|**
Intercept						
	-2.5102	0.0280	-2.5651	-2.4552	-89.52	<.0001
Wave						
Non-Pioneer	ref	ref	ref	ref	ref	ref
Pioneer	-0.0035	0.0181	-0.0389	0.0319	-0.19	0.8466
Time						
	0.0737	0.0056	0.0628	0.0846	13.26	<.0001
Interaction Time*Wave						
	-0.0252	0.0165	-0.0577	0.0072	-1.53	0.1272
Age Group						
00-19	ref	ref	ref	ref	ref	ref
20-39	-0.1454	0.0117	-0.1683	-0.1225	-12.45	<.0001
40-59	-0.0638	0.0131	-0.0894	-0.0382	-4.88	<.0001
60-79	0.6936	0.0149	0.6644	0.7228	46.57	<.0001
80+	1.8742	0.0155	1.8439	1.9045	121.17	<.0001
Sex						
Male	ref	ref	ref	ref	ref	ref

For peer review only

For peer review only - http://bmjopen.bmj.com/site/about/guidelines.xhtml

Female	0.0978	0.0031	0.0918	0.1039	31.74	<.0001
Index of Multiple Deprivation Decile						
1	ref	ref	ref	ref	ref	ref
2	-0.0927	0.0330	-0.1573	-0.0280	-2.81	0.0049
3	-0.1168	0.0342	-0.1838	-0.0498	-3.42	0.0006
4	-0.1692	0.0345	-0.2368	-0.1016	-4.90	<.0001
5	-0.2203	0.0339	-0.2868	-0.1538	-6.49	<.0001
6	-0.2143	0.0316	-0.2762	-0.1523	-6.78	<.0001
7	-0.3083	0.0314	-0.3699	-0.2467	-9.81	<.0001
8	-0.3066	0.0328	-0.3709	-0.2422	-9.33	<.0001
9	-0.3718	0.0272	-0.4251	-0.3186	-13.68	<.0001
10	-0.4264	0.0288	-0.4828	-0.3700	-14.82	<.0001

For peer review only

For peer review only - http://bmjopen.bmj.com/site/about/guidelines.xhtml

STROBE Statement—checklist of items that should be included in reports of observational studies

	Item No	Recommendation	Page No
**Title and abstract**	1	(*a*) Indicate the study's design with a commonly used term in the title or the abstract	1
		(*b*) Provide in the abstract an informative and balanced summary of what was done and what was found	2
**Introduction**			
Background/rationale	2	Explain the scientific background and rationale for the investigation being reported	5-6
Objectives	3	State specific objectives, including any prespecified hypotheses	6
**Methods**			
Study design	4	Present key elements of study design early in the paper	6
Setting	5	Describe the setting, locations, and relevant dates, including periods of recruitment, exposure, follow-up, and data collection	6-7
Participants	6	(*a*) *Cohort study*—Give the eligibility criteria, and the sources and methods of selection of participants. Describe methods of follow-up<br>*Case-control study*—Give the eligibility criteria, and the sources and methods of case ascertainment and control selection. Give the rationale for the choice of cases and controls<br>*Cross-sectional study*—Give the eligibility criteria, and the sources and methods of selection of participants	6-7
		(*b*) *Cohort study*—For matched studies, give matching criteria and number of exposed and unexposed<br>*Case-control study*—For matched studies, give matching criteria and the number of controls per case	-
Variables	7	Clearly define all outcomes, exposures, predictors, potential confounders, and effect modifiers. Give diagnostic criteria, if applicable	6-7
Data sources/ measurement	8*	For each variable of interest, give sources of data and details of methods of assessment (measurement). Describe comparability of assessment methods if there is more than one group	*6-7*
Bias	9	Describe any efforts to address potential sources of bias	7-8
Study size	10	Explain how the study size was arrived at	-
Quantitative variables	11	Explain how quantitative variables were handled in the analyses. If applicable, describe which groupings were chosen and why	7-8
Statistical methods	12	(*a*) Describe all statistical methods, including those used to control for confounding	7-8
		(*b*) Describe any methods used to examine subgroups and interactions	-
		(*c*) Explain how missing data were addressed	-
		(*d*) *Cohort study*—If applicable, explain how loss to follow-up was addressed<br>*Case-control study*—If applicable, explain how matching of cases and controls was addressed<br>*Cross-sectional study*—If applicable, describe analytical methods taking account of sampling strategy	-
		(*e*) Describe any sensitivity analyses	-

Continued on next page

For peer review only - http://bmjopen.bmj.com/site/about/guidelines.xhtml

**Results**			
Participants	13*	(a) Report numbers of individuals at each stage of study—eg numbers potentially eligible, examined for eligibility, confirmed eligible, included in the study, completing follow-up, and analysed	9-10
		(b) Give reasons for non-participation at each stage	-
		(c) Consider use of a flow diagram	-
Descriptive data	14*	(a) Give characteristics of study participants (eg demographic, clinical, social) and information on exposures and potential confounders	9
		(b) Indicate number of participants with missing data for each variable of interest	-
		(c) *Cohort study*—Summarise follow-up time (eg, average and total amount)	-
Outcome data	15*	*Cohort study*—Report numbers of outcome events or summary measures over time	-
		Case-control study—Report numbers in each exposure category, or summary measures of exposure	-
		Cross-sectional study—Report numbers of outcome events or summary measures	*9-10*
Main results	16	(*a*) Give unadjusted estimates and, if applicable, confounder-adjusted estimates and their precision (eg, 95% confidence interval). Make clear which confounders were adjusted for and why they were included	9-11
		(*b*) Report category boundaries when continuous variables were categorized	9-11
		(*c*) If relevant, consider translating estimates of relative risk into absolute risk for a meaningful time period	-
Other analyses	17	Report other analyses done—eg analyses of subgroups and interactions, and sensitivity analyses	-
**Discussion**			
Key results	18	Summarise key results with reference to study objectives	11
Limitations	19	Discuss limitations of the study, taking into account sources of potential bias or imprecision. Discuss both direction and magnitude of any potential bias	11-12
Interpretation	20	Give a cautious overall interpretation of results considering objectives, limitations, multiplicity of analyses, results from similar studies, and other relevant evidence	12-14
Generalisability	21	Discuss the generalisability (external validity) of the study results	12-14
**Other information**			
Funding	22	Give the source of funding and the role of the funders for the present study and, if applicable, for the original study on which the present article is based	3

*Give information separately for cases and controls in case-control studies and, if applicable, for exposed and unexposed groups in cohort and cross-sectional studies.

Note: An Explanation and Elaboration article discusses each checklist item and gives methodological background and published examples of transparent reporting. The STROBE checklist is best used in conjunction with this article (freely available on the Web sites of PLoS Medicine at http://www.plosmedicine.org/, Annals of Internal Medicine at http://www.annals.org/, and Epidemiology at http://www.epidem.com/). Information on the STROBE Initiative is available at www.strobe-statement.org.

For peer review only - http://bmjopen.bmj.com/site/about/guidelines.xhtml

BMJ Open

Area level impacts on emergency hospital admissions of the Integrated Care and Support Pioneer Programme in England: difference-in-differences analysis

Journal:	*BMJ Open*
Manuscript ID	bmjopen-2018-026509.R1
Article Type:	Research
Date Submitted by the Author:	17-Jun-2019
Complete List of Authors:	Keeble, Eilís; The Nuffield Trust<br>Bardsley, M; The Health Foundation<br>Durand, Mary Alison; London School of Hygiene and Tropical Medicine, Policy Innovation and Evaluation Research Unit, Department of Health Services Research and Policy<br>Hoomans, Ties ; London School of Hygiene and Tropical Medicine, Policy Innovation and Evaluation Research Unit, Department of Health Services Research and Policy<br>Mays, Nicholas; London School of Hygiene and Tropical Medicine, Policy Innovation and Evaluation Research Unit, Department of Health Services Research and Policy
**Primary Subject Heading**:	Health services research
Secondary Subject Heading:	Health policy
Keywords:	Quality in health care < HEALTH SERVICES ADMINISTRATION & MANAGEMENT, STATISTICS & RESEARCH METHODS, Organisation of health services < HEALTH SERVICES ADMINISTRATION & MANAGEMENT, Health policy < HEALTH SERVICES ADMINISTRATION & MANAGEMENT

SCHOLARONE™ Manuscripts

Area level impacts on emergency hospital admissions of the Integrated Care and Support Pioneer Programme in England: difference-in-differences analysis

Corresponding author: Eilís Keeble, The Nuffield Trust, 59 New Cavendish Street, London, W1G 7LP, United Kingdom, email: eilis.keeble@nuffieldtrust.org.uk, phone: 0207 631 8450

Martin Bardsley, The Health Foundation (Formerly of The Nuffield Trust), London, United Kingdom

Mary Alison Durand, Policy Innovation and Evaluation Research Unit, Department of Health Services Research and Policy, London School of Hygiene and Tropical Medicine, London, United Kingdom

Ties Hoomans, Policy Innovation and Evaluation Research Unit, Department of Health Services Research and Policy, London School of Hygiene and Tropical Medicine, London, United Kingdom

Nicholas Mays, Policy Innovation and Evaluation Research Unit, Department of Health Services Research and Policy, London School of Hygiene and Tropical Medicine, London, United Kingdom

Word Count: 3,151

For peer review only

ABSTRACT

Objective

To examine whether any differential change in emergency admissions could be attributed to integrated care by comparing Pioneer and non-Pioneer populations from a pre-Pioneer baseline period (April 2010 to March 2013) over two follow-up periods: to 2014/15 and to 2015/16.

Design

Difference-in-differences analysis of emergency hospital admissions from English Hospital Episode Statistics (HES).

Setting

Local authorities in England classified as either Pioneer or non-Pioneer.

Participants

Emergency admissions to all NHS hospitals in England with local authority determined by area of residence of the patient.

Intervention

Wave 1 of the Integrated Care and Support Pioneer Programme announced in November 2013.

Primary Outcome Measure

Change in hospital emergency admissions.

Results

The increase in the Pioneer emergency admission rate from baseline to 2014/15 was smaller at 1.98 per cent and significantly different from that of the non-Pioneers at 4.85 per cent (p=0.0395). The increase in the Pioneer emergency admission rate from baseline to 2015/16 was again smaller than for the non-Pioneers but the difference was not statistically significant (p=0.1905).

Conclusions

It is ambitious to expect unequivocal changes in a high level and indirect indicator of health and social care integration such as emergency hospital admissions to arise as a result of the

For peer review only

changes in local health and social care provision across organisations brought about by the Pioneers in their early years and we should treat any sign that the Pioneers have had such an impact with caution. Nevertheless, there does seem to be an indication from the current analysis that there were some changes in hospital use associated with the first year of Pioneer status that are worthy of further exploration.

STRENGTHS AND LIMITATIONS OF THIS STUDY

1. This study adds to the evidence of the impact of system-wide approaches to integrating health and social care, like the Pioneer Programme, using advanced statistical methods todetermine whether the Pioneers reduced emergency admissions.
2. Reducing emergency admissions is often cited as a key goal of new integrated models of care and the Hospital Episode Statistics provide a continuously collected person level dataset to enable tracking of changes over time at small area level.
3. Analysing the Pioneer sites collectively ensured the inclusion of a diverse range of areas which were unlikely to be systematically different at baseline from the non-Pioneers.
4. It is difficult to find a true counter-factual population to compare with the Pioneers as many other initiatives related to health and social care integration had been developed in other areas of the country previously and/or were being implemented almost simultaneously.
5. The Pioneers invested in a collection of health and social care integration strategies and interventions; identifying the causes and effects of these specific initiatives would require detailed local primary data collection but this analysis focuses on the overall impact of the Pioneers as a national policy initiative.

KEYWORDS

Organisation of health services, quality in health care, health policy, statistics and research methods

For peer review only

INTRODUCTION

In November 2013, the Integrated Care and Support Pioneer programme was initiated in England. The programme aimed to promote integration between the separate local health and social care systems in England by facilitating these systems to develop and implement new ways of working together with the objective of meeting people's needs better and improving service users' experience of care .[1]

In the first wave of the programme the English Department of Health (DH) (now Department of Health and Social Care (DHSC)) selected 14 Pioneer areas from a round of competitive applications, that were identified as the "most ambitious and visionary" in their plans for health and social care system integration.[2] Each Pioneer was given access to limited support and expertise over a five-year period and a one-off fund of £90,000 to help with initial development. A second wave of 11 Pioneer areas was subsequently announced in January 2015. These are excluded from the present analysis as there are insufficient time points available currently for an interpretable trend analysis.

Integration in the Pioneer areas has taken on different forms. As Erens and colleagues noted, "*What it meant to be 'a Pioneer' varied between sites and between individuals within sites. At various times it was apparent that Pioneer status meant one or more of the following:*

- *a 'badge' for a locality signifying national recognition of innovation and progress in integrating care*
- *an enabler of the existing local plan for transformation*
- *a particular governance arrangement, for example a Board that brought all system leaders and their organisations around the table*
- *a collection of discrete workstreams, characteristically covering a combination of different groups of users and infrastructure projects (for example, information sharing, workforce development, etc.)*
- *a specific new integrated service, such as a frailty service*
- *an ethos or way of thinking about and providing care, rather than a specific plan or set of initiatives"*[3]

For peer review only

Some of the Pioneers planned to focus on specific populations. Of these, the most common were older people, people with long term conditions and people at high risk of hospitalisation. Broadly, however, the Pioneers shared the same vision for the future of the health and social care system by seeking to create a 'whole system' of integrated care involving all local bodies and professional groups organised around the needs of individuals and their informal carers which set them apart from the rest of England.[4]

All but one (Stoke and North Staffordshire) of the Wave 1 Pioneers stated that reducing emergency admissions was an aim or an expected outcome of integration in their original bid. Risk stratification with targeted interventions and introducing preventive strategies to avoid the need for acute hospitalisation were listed as activities to achieve this goal (see supplementary material). The focus on reducing emergency hospital care use was given still greater emphasis by the Pioneers as financial austerity bit more deeply into local health care budgets after 2013.[3]

As a consequence of the focus on emergency hospital care as a costly service, the success of integrated care initiatives has often been presented, at least in part, in terms of their ability to reduce the need for emergency hospital admissions and to reduce emergency admission rates.[5] Reducing emergency admission rates has been a feature of English health policy over the past decade and continues to be one of the most commonly used measures of success for system change initiatives.[6–8] To date, however, there has been little evidence of initiatives successfully reducing emergency admissions.[9–11]

This paper presents new evidence on the effect of the Pioneer programme on emergency admissions. We investigate changes in the emergency admissions to hospitals of patients across England following the implementation of the programme in 2013. The analysis is part of a wider programme of evaluation of the Pioneers (http://piru.lshtm.ac.uk/projects/current-projects/integrated-care-pioneers-evaluation.html). Though it is not possible to identify precisely which elements of the programme, if any, led to any differential change observed (since the Pioneers were not working from an agreed template), such an analysis can be justified as a necessary step in understanding the impacts of a major initiative such as the integrated care Pioneer programme, especially since it had much in common with successive initiatives such as the

For peer review only

New Care Model Vanguards and the current focus on Integrated Care Systems (ICSs).[12-13] The underlying hypothesis is that the cumulative effect of the specific initiatives embedded in each Pioneer programme would bring about sufficient change in emergency hospital care use as to be detectable at the level of the whole population of the Pioneers.

METHODS

To examine whether differential change in emergency admissions could be attributed to Pioneer status a difference-in-differences approach was used. Difference-in-differences measures the effect of the intervention (the Pioneer programme) by looking at the change in emergency admissions between the two time points in the two groups and quantifies whether or not the population within the Pioneer programme experiences a change that is significantly different to the comparison group, the non-Pioneers.

Data Sources

We used inpatient Hospital Episode Statistics (HES) to identify all emergency admissions to NHS hospitals in Pioneer and non-Pioneer areas across England. HES is collated by NHS Digital and is a pseudonymous patient level dataset that records basic features of admissions to hospital including: patient age, sex, admission date and emergency admission indicator (admission methods starting with "2").[14]

To be able to compare emergency admission rates between areas (Pioneer/non-Pioneer), we also obtained information on key local authority level factors determining local population health and care needs:

- Demographic composition (age and sex), from the Office for National Statistics (ONS).[15]
- Deprivation decile, from the 2015 Index of Multiple Deprivation.[16]

Defining Pioneer Areas

The Pioneer areas did not all map neatly to a single set of health or local government administrative boundaries. After consultation with each Pioneer, they were mapped to the local authorities which most closely aligned with the intervention area (see supplementary material for lookup table). Local authority boundaries were used instead of health boundaries as the population denominators could be linked over a longer period. A wider breadth of data

For peer review only

is available for this boundary which is being used in other parts of the evaluation, for example social care data.

The local authorities which were linked to the second wave of Pioneers, initiated in January 2015, were excluded from all analyses and not included in either the Pioneer or non-Pioneer populations. Non-Pioneer areas were defined as any local authority that was not a first or second wave Pioneer.

Defining Time Periods

A baseline period before Pioneer programme implementation of April 2010 to March 2013 was compared to two follow-up periods: April 2014 to March 2015 (2014/15) and April 2015 to March 2016 (2015/16). The period April 2013 to March 2014 was excluded as this encompassed the call for applications to the programme (May 2013) and the announcement of the sites (November 2013).

Outcome

Our primary outcome was the average percentage difference in rates of emergency hospital admissions per 100,000 between baseline and follow-up (2014/15 or 2015/16) for the study groups (Pioneers/non-Pioneers). Area-level rates were calculated as the total number of emergency admissions over each time period divided by the mid-year population for each group. Admissions were derived by month and local authority of residence. They were adjusted for deprivation decile, age group (0-19, 20-39, 40-59, 60-79, 80+) and sex. The English age, sex and deprivation decile structure was used as the reference population for each local authority for the initial analysis. The secondary outcome was the difference in average percentage change in the rates over time between the Pioneers and non-Pioneers.

Statistical Analyses

An initial difference-in-differences comparison was performed by looking at the change in the adjusted emergency admission rate for the Pioneers and non-Pioneers. Percentage differences between the baseline period and the two follow-up time points of 2014/15 and 2015/16 were calculated, along with the difference between these.

To determine whether the change in emergency admissions in the Pioneers was significantly different from the change in the non-Pioneers, we performed difference-in-differences

For peer review only

regression analysis. We estimated negative binomial regression models for count data adjusting for age, sex and deprivation decile. Poisson models were first attempted but the data were over-dispersed and unsuitable. Each regression model included a continuous local authority population size exposure variable, a binary Pioneer status term (Pioneer/non-Pioneer), a binary time term (baseline/follow-up), a difference-in-differences term (Pioneer status*time) and covariate terms. We obtained robust standard error estimates adjusting for clustering of the repeated measures from each local authority. Significance was assessed at $p<0.05$. SAS 9.4® was used for all analyses.

For peer review only

Difference-in-Differences Estimation Validation Tests

To validate our difference-in-difference estimations, we tested the following assumptions:

1. That areas were not selected into the Programme based on emergency admission rates at baseline, by comparing baseline emergency admissions and demographics of the Pioneer and non-Pioneers.
2. That changes in emergency admission rates over time would be the same for both the Pioneer and non-Pioneer areas in the absence of the Pioneer programme, by comparing adjusted emergency admission rates for the Pioneers and non-Pioneers over the baseline period. These were compared graphically and statistically using a linear time trend of month in the baseline period interacted with Pioneer status controlling for age, sex and deprivation decile.

Sensitivity Analyses

We examined sensitivity of the main findings to excluding Stoke and North Staffordshire Pioneer from our analyses and to using individual years for the baseline period (see supplementary material). Stoke and North Staffordshire had a unique target population and no focus on reducing emergency admissions. As the baseline period covered three years, each individual baseline year was also compared to the first follow-up time point.

Patient and Public Involvement

Patient and public representatives are involved in the wider evaluation of which this analysis forms a part and were involved in the selection and peer review of the initial proposal on which this analysis is based.

RESULTS

Baseline Characteristics

The characteristics of the Pioneers and non-Pioneers during the baseline period of April 2010 to March 2013 are summarised in Table 1. The Pioneers consisted of 49 local authorities and encompassed 17 percent of the English population in the baseline period.[15] The proportions of the population aged 65 and over, or female, were similar between the two groups. Area level deprivation in the Pioneers was slightly higher than in the non-Pioneers.

Table 1 Baseline Characteristics of the Pioneer and non-Pioneer populations

Characteristic	Pioneers First Wave (n = 14)*	Non-Pioneers
Number of Local Authorities	49	244
Average Yearly Population at Baseline	9,083,051	37,137,613
Proportion Population Under 20	24%	24%
Proportion Population Aged 65+	16%	17%
Proportion Population Female	50%	50%
Average Local Authority IMD Score (2015)	21.1	18.7

* 11 second wave Pioneers and 33 associated local authorities were excluded from the analyses

Trend Analysis

Figure 1 shows the adjusted monthly emergency admission rates for the Pioneers and non-Pioneers between April 2010 and March 2016. On visual inspection, the trends in the baseline period overlap which indicates that trend bias should have limited impact on the difference-in-differences analysis. A statistical test of the trends in the baseline period also indicated limited trend bias (p=0.7378).

Difference-in-Differences

Between the baseline period and the first follow-up period (2014/15) average emergency admission rates decreased by 0.42% for the Pioneer and increased by 3.46% for the non-Pioneers, with a difference-in-differences of 3.89% (see Table 2). When the baseline was compared to the second follow-up period (2015/16), the Pioneers still had a lower increase at 2.23% but the difference compared to the non-Pioneers was smaller at 3.23%.

For peer review only

Table 2 Emergency admission rates for Pioneers and Non-Pioneers (adjusted for age, sex and deprivation decile) at baseline and follow-up, with percentage differences compared to baseline and difference in difference between non-Pioneer and Pioneer

	Emergency Admission Rate (per 100,000 population)			Percentage Difference		Difference-in-Differences*	
	Baseline	2014/15	2015/16	2014/15	2015/16	2014/15	2015/16
Non-Pioneer	9,942	10,287	10,485	3.46%	5.46%	3.89%	3.23%
Pioneer	9,948	9,906	10,170	-0.42%	2.23%		

** Difference between the non-Pioneer and Pioneer percentage difference, positive value indicates non-Pioneer change is greater*

Trends for the individual Pioneers varied. For example, half the Pioneers had a percentage increase in their emergency admission rates between baseline and 2014/15, while the percentage difference for the Pioneers as a whole was a slight decrease (see Table 3). There was also variation within Pioneers (see supplementary material). For example, the constituent local authorities comprising the Waltham Forest, East London and City Pioneer had declines in emergency admission rates ranging from -10.45% (Tower Hamlets) to -1.64% (Newham) between baseline and 2014/15, while the overall percentage difference was -9.85%.

Table 3 Emergency admission rates for individual pioneers (adjusted for age, sex and deprivation decile) at baseline and follow-up, with percentage differences compared to baseline

Pioneer (Number of LAs)	Emergency Admission Rate (per 100,000 population)			Percentage Difference to Baseline	
	Baseline	2014/15	2015/16	2014/15	2015/16
Barnsley (1)	10,992	11,769	12,325	7.07%	12.13%
Cheshire (2)	11,259	12,160	12,459	8.00%	10.65%
Cornwall and Isles of Scilly (2)	8,170	8,061	8,193	-1.33%	0.29%
Greenwich (1)	8,168	8,226	9,513	0.71%	16.47%
Islington (1)	6,324	6,320	6,096	-0.06%	-3.60%
Kent (12)	9,349	10,033	10,009	7.32%	7.06%
Leeds (1)	11,399	9,605	10,155	-15.74%	-10.91%
North West London (8)	8,922	8,665	8,812	-2.87%	-1.23%
South Devon and Torbay (3)	7,415	7,630	8,803	2.90%	18.72%
South Tyneside (1)	11,153	10,445	11,150	-6.35%	-0.03%
Southend (1)	9,243	10,397	10,224	12.49%	10.61%
Stoke and North Staffordshire (7)	9,949	10,253	10,611	3.06%	6.66%
Waltham Forest, East London and City (3)	9,184	8,657	8,279	-5.73%	-9.85%
Worcestershire (6)	9,018	8,817	9,006	-2.23%	-0.13%

For peer review only

Difference-in-Differences Regression

After adjusting for age, sex and deprivation, the difference-in-differences regression analysis showed that the change in emergency admission rates in the Pioneers between baseline and 2014/15 was smaller and significantly different from that of the non-Pioneers (p=0.0394) (see Table 4). The Pioneer emergency admission rate increased by 1.98 per cent compared to 4.85 per cent in the non-Pioneers. When comparing baseline with 2015/16, the analysis still indicated that the change in emergency admissions for the Pioneers was smaller at 4.07 per cent compared to 6.36 percent for the non-Pioneers but the difference was not statistically significant (p=0.1905).

Table 4 Difference in difference model coefficients and percentage difference in emergency admissions for Pioneers and Non-Pioneers, adjusted for age, sex and l deprivation

	2014/15	2015/16
**Model Coefficients** (p value)		
Intercept	-5.4234 (<0.0001)	-5.4259 (<0.0001)
Non-Pioneer/Pioneer	-0.0044 (0.8169)	-0.0043 (0.8190)
Baseline/Follow-Up	0.04173 (<0.0001)	0.0617 (<0.0001)
Interaction	-0.0278 (0.0394)	-0.0218 (0.1905)
**Percentage Difference** [95% confidence interval]		
Non-Pioneer	4.85 [3.68,6.03]	6.36 [5.05,7.70]
Pioneer	1.98 [-0.37,4.38]	4.07 [1.03,7.20]

DISCUSSION

The Integrated Care and Support Pioneers represent one important example of how English health and social care services have been exploring new ways of working across organisational boundaries. The aims of the individual Pioneers varied,[3] but most had a common interest in providing care and support that was intended to reduce the need for urgent care services and lead to a reduction in emergency hospital admissions. After comparing changes in emergency admissions from a three-year pre-Pioneer baseline period between Pioneer populations and non-Pioneer populations, we found a lower increase in emergency admissions for the Pioneers than the non-Pioneers. This lower increase was statistically significant for the comparison between baseline and 2014/15 (p=0.0394) but not for the comparison between baseline and 2015/16 (p=0.1905).

For peer review only

This type of population level analysis can help provide some independent evidence of the likely scale of changes within an area associated with integrated care initiatives and curb some of the more zealous rhetoric for or against integrated health and social care, and related changes in service delivery. Looking at emergency admission data on this scale means the outcome of interest is based on a relatively large number of events and continuously collected data – making them useful as a measure of potential programme impact. This is in contrast to a range of other potential measures of health and social care integration at community level that are likely to be less sensitive to short term change such as annual patient experience surveys. The size and range of geographical areas covered by both the Pioneers and non-Pioneers along with their socio-demographic similarities should mean that differences in factors such as supply of social care services or acute hospital beds and the process of collecting data are unlikely to be systematically different between the two groups beyond any changes associated with Pioneer status.

It would be beneficial to track emergency admissions for more than two years to measure the impact of policy initiatives such as the Pioneers more definitively. However, during the life of the Pioneer programme, there were parallel changes in the wider policy context both in terms of specific health and care integration policies such as the Better Care Fund,[17] the overall level of funding for both health and social care in a period of unprecedented financial austerity and,[18] from 2015 onwards, the New Care Model Vanguards.[19] In particular, the Vanguards' approach to improving care coordination had much in common with the Pioneers. This means that the ideas behind integration that prompted the Pioneers and the types of interventions that they developed are no longer (if they ever were) unique to these areas and are being implemented across the country. Therefore, a true counter-factual population is difficult to find. This may, in part, explain why the difference between the Pioneers and non-Pioneers reduced between baseline and 2015/16 compared with baseline and 2014/15 as the behaviour of the non-Pioneers becomes increasingly similar to the Pioneers.[20] This is in part to be expected as disseminating learning from the Pioneers was actively encouraged as part of the programme.

In addition to the difficulties of finding a counterfactual over the life-time of the Pioneers, it was not the first programme to focus on health and social care integration in England. One such previous initiative was the Integrated Care Pilots. While an effect on emergency

For peer review only

admissions was not found for this programme, it can't be ruled out that these pilots have had a legacy impact on emergency admissions.[21] It should therefore be note that, three of the Pioneers overlap with areas that were previously Integrated Care Pilots (Cornwall, Torbay and Tower Hamlets) and therefore, may have had a focus on integration for longer than some other Pioneers. This may in part explain the steady declines in emergency admissions seen in Tower Hamlets and to a lesser extent Cornwall. Seven of the Integrated Care Pilots also covered areas which were not Pioneers and therefore, the impact of the Pioneers in contrast to these may be reduced.

A more detailed understanding of the impacts of the Pioneers would be gained with a targeted analytical approach using information on the specific initiatives implemented in each Pioneer and data on the exact populations in receipt of these initiatives (this is being attempted in another component of the Pioneer evaluation). While this might yield gains in terms of causal inference in that changes could potentially be attributed to a specific set of local actions, such an analysis might lose the ability to assess the impact of change across a system and an entire population. This is important to note as the Pioneers were intended to be a complex mix of specific service changes and initiatives, supported by a wider pattern of infrastructural changes at the level of the local health and social care system.

Other studies have looked at schemes with an aspiration to reduce the need for urgent hospital care through better coordinated health and care services, and with an emphasis on preventing admissions. Success is typically assessed in terms of reduction in emergency hospital admissions and various previous evaluations show that this has been difficult to achieve.[9–11] Despite the intense policy interest in how different forms of service delivery can reduce emergency admissions, there are few, if any, studies showing unequivocal change in the direction desired. Against this backdrop, the modest changes observed across the 14 wave one Pioneer areas in their first two years look promising. However, when exploring the extent to which the observed changes are likely to be related causally to Pioneer status, it should be noted that:

a. the effect appears to be temporary: and as such the effect may have been linked to changes that took place in the early stages of the Pioneers or pre-Pioneer but

For peer review only

were not sustained; or the non-Pioneer areas introduced changes which have subsequently reduced the difference between them and the Pioneers; and

b. the changes in emergency admissions were not shown in all places and even varied between local authority areas within the same Pioneer.

Conclusion

It is ambitious to expect unequivocal changes in a single high level and indirect indicator of health and care integration such as emergency hospital admissions to arise as a result of changes in local health and care provision across organisations brought about by the Pioneers in their early years. We should treat any signs that the Pioneers have had such an impact with caution. Nevertheless, our analysis does seem to provide some evidence that there were some changes in hospital use associated with the first year of Pioneer status that are worthy of further exploration. At the very least, this analysis shows that Pioneer status does not seem to have been associated with a relative deterioration in performance in terms of emergency hospital use.

FIGURE LEGEND

Figure 1 Emergency admission rate for Pioneers and Non-Pioneers by month adjusted for age, sex and deprivation decile (Pioneer intervention introduced in shaded area)

AUTHOR CONTRIBUTIONS

EK undertook the analysis and with MB drafted the initial paper. MB and NM contributed to design of the study. EK, MB, MAD, TH and NM contributed to interpretation of findings and revisions of the paper.

ACKNOWLEDGEMENTS

This work uses data provided by patients and collected by the NHS as part of their care and support. Read more at https://www.nuffieldtrust.org.uk/about/corporate-policies#information-security-and-data.

Hospital Episode Statistics data (year range 2007/08–2015/16) Copyright © (2018), NHS Digital. Re-used with the permission of NHS Digital. All rights reserved.

For peer review only

FUNDING

This study is funded by the NIHR Policy Research Programme (*Evaluation of the Integrated Care and Support Pioneers Programme in the context of new funding arrangements for integrated care in England* (2015-2020), PR-R10-0514-25001). The views expressed are those of the authors and are not necessarily those of the NIHR or the Department of Health and Social Care.

COMPETING INTERESTS

This study is funded by the NIHR Policy Research Programme (*Evaluation of the Integrated Care and Support Pioneers Programme in the context of new funding arrangements for integrated care in England* (2015-2020), PR-R10-0514-25001). The views expressed are those of the authors and are not necessarily those of the NIHR or the Department of Health and Social Care.

DATA AVAILABILITY

This study used Hospital Episode Statistics data obtained from NHS Digital under a data sharing agreement and are reused with their permission. Hospital Episode Statistics data may be obtained from NHS Digital under a similar process but we are unable to share it per the terms of our agreement.

For peer review only

REFERENCES

[1] NHS England. Integrated care pioneers announced. 2013. Available: https://www.england.nhs.uk/2013/11/interg-care-pioneers/. [Accessed: 01-Aug-2018].

[2] Department of Health. Letter inviting expressions of interest for health and social care integration "Pioneers". 2013. Available: https://www.gov.uk/government/publications/social-care-integration-pioneers. [Accessed: 01-Aug-2018].

[3] Erens B, Wistow G, Mounier-Jack S, et al. Early evaluation of Integrated Care and Support Pioneers Programme: Final Report. Policy Innovation Research Unit. 2015. Available: http://piru.lshtm.ac.uk/assets/files/Early_evaluation_of_IC_Pioneers_Final_Report.pdf . [Accessed: 01-Aug-2018].

[4] National Collaboration for Integrated Care and Support. Integrated Care and Support: Our Shared Commitment. 2013. Available: https://assets.publishing.service.gov.uk/government/uploads/system/uploads/attachment_data/file/198748/DEFINITIVE_FINAL_VERSION_Integrated_Care_and_Support_-_Our_Shared_Commitment_2013-05-13.pdf. [Accessed: 01-Aug-2018].

[5] Raleigh V, Bardsley M, Smith P, et al. Integrated care and support Pioneers: Indicators for measuring the quality of integrated care: Final report. Policy Innovation Research Unit. 2014. Available: http://piru.lshtm.ac.uk/assets/files/IC_and_support_Pioneers-Indicators.pdf. [Accessed: 01-Aug-2018].

[6] National Audit Office. Emergency admissions to hospital: managing the demand. National Audit Office. 2013. Available: https://www.nao.org.uk/wp-content/uploads/2013/10/10288-001-Emergency-admissions.pdf. [Accessed: 01-Aug-2018].

[7] Wallace E, Smith SM, Fahey T, et al. Reducing emergency admissions through community based interventions. *BMJ* 2016;352:h6817 doi:10.1136/bmj.h6817 [published Online first: 28 January 2016].

[8] Lloyd T, Wolters A, Steventon A. The impact of providing enhanced support for care home residents in Rushcliffe. The Health Foundation. 2017. Available: https://www.health.org.uk/publication/impact-enhanced-support-rushcliffe. [Accessed: 01-Aug-2018].

For peer review only

[9] Bardsley M, Steventon A, Smith J, et al. Evaluating integrated and community-based care: How do we know what works? The Nuffield Trust. 2013. Available: https://www.nuffieldtrust.org.uk/files/2017-01/evaluating-integrated-community-care-web-final.pdf. [Accessed: 01-Aug-2018].

[10] Roland M, Lewis R, Steventon A, et al. Case management for at-risk elderly patients in the English integrated care pilots: observational study of staff and patient experience and secondary care utilisation. *Int J Integr Care* 2012;12(5) http://doi.org/10.5334/ijic.850.

[11] Purdy S, Paranjothy S, Huntley A, et al. Interventions to reduce unplanned hospital admission: a series of systematic reviews. National Institute for Health Research. 2012. Available: http://www.bristol.ac.uk/media-library/sites/primaryhealthcare/migrated/documents/unplannedadmissions.pdf. [Accessed: 01-Aug-2018].

[12] NHS England. New Care Models: Vanguards – developing a blueprint for the future of the NHS and care services. 2016. Available: https://www.england.nhs.uk/wp-content/uploads/2015/11/new_care_models.pdf [Accessed: 11-Jun-2019]

[13] NHS England. Breaking down barriers to better health and care. 2019. Available: https://www.england.nhs.uk/wp-content/uploads/2019/04/breaking-down-barriers-to-better-health-and-care-march19.pdf [Accessed: 11-Jun-2019]

[14] NHS Digital. Hospital Episode Statistics data 2007/08-2015/16.

[15] Office for National Statistics. Population estimates 2007-2015. Available: https://www.ons.gov.uk/peoplepopulationandcommunity/populationandmigration/populationestimates. [Accessed: 14-May-2018].

[16] Ministry of Housing, Communities and Local Government. English indices of deprivation 2015. Available: https://www.gov.uk/government/statistics/english-indices-of-deprivation-2015. [Accessed: 12-Jun-2018].

[17] NHS England. Better Care Fund. Available: https://www.england.nhs.uk/ourwork/part-rel/transformation-fund/bcf-plan/. [Accessed: 14-May-2018].

[18] Robertson R, Wenzel L, Thompson J, et al. Understanding NHS financial pressures: How are they affecting patient care? The Kings Fund. 2017.

For peer review only

[19] NHS. NHS Five Year Forward View. 2014. Available: https://www.england.nhs.uk/wp-content/uploads/2014/10/5yfv-web.pdf. [Accessed: 01-Aug-2018].

[20] Chen YF, Hemming K, Stevens AJ, et al. Secular trends and evaluation of complex interventions: the rising tide phenomenon. *BMJ Qual Saf 2016;*25(5)303–310 doi: 10.1136/bmjqs-2015-004372 [published Online first: 06 Oct 2015].

[21] RAND Europe and Ernst & Young LLP. National Evaluation of the Department of Health's Integrated Care Pilots: Final Report. 2012. Available: https://assets.publishing.service.gov.uk/government/uploads/system/uploads/attachment_data/file/215103/dh_133127.pdf [Accessed: 14-Jun-2019]

For peer review only

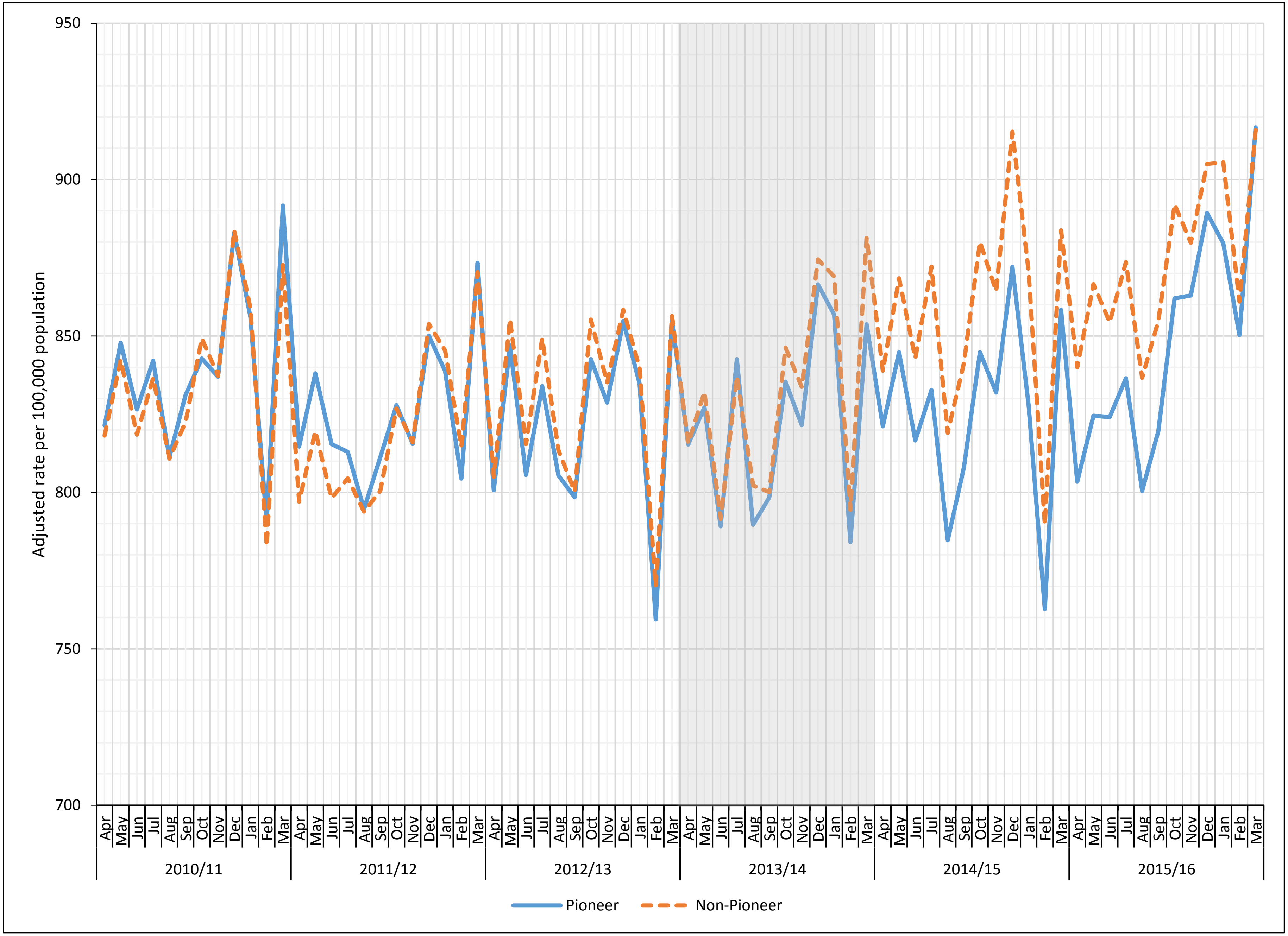

For peer review only - http://bmjopen.bmj.com/site/about/guidelines.xhtml

SUPPLEMENTARY MATERIAL

1. Target populations, integration mechanisms, activities and emergency admission focus of Wave 1 Pioneers
2. Local Authority to Pioneer Lookup
3. Emergency admission rates for individual local authorities within Pioneers
4. Sensitivity analysis: excluding Stoke and North Staffordshire Pioneer
5. Sensitivity analysis: individual baseline years compared to 2014/15

For peer review only

1. Target populations, integration mechanisms, activities and emergency admission focus of Wave 1 Pioneers

Pioneer	Target Population(s)	Integration Mechanisms	Examples of Specific Activities	Reduced emergency admissions
Barnsley	Whole population with focus on children and families	Integrated programme boards;<br>Adopting a citizenship approach at all levels of the community to move from intervention to prevention;<br>Provision of information, advice, and signposting through services such as telecare.	Universal information and advice service;<br>Be Well Barnsley: community orientated prevention;<br>Immediate care review;<br>Integrated personal budgets	Aim
Cheshire	Older adults with chronic conditions; individuals with mental health issues; complex needs families	Integration commissioning through a joint governance board, redesigned care and intervention pathways and joint investment plan involving the voluntary and community sectors;<br>Integration of care management and investment into integration enablers, such as shared records and telehealth services.	New funding/contracting model for the acute sector and community care;<br>Hospital at home;<br>Partnership working between Health Care Management Financial Association, Health Care Services and Net Orange to reduce hospital admissions;	Outcome
Cornwall and Isles of Scilly	People who are at risk of becoming high users of health and social care services	Integrated leadership through shared goals and performance measures, information sharing and a new funding contract;<br>Investment in telehealth;<br>Integrated care through multi-disciplinary teams, workforce development, information sharing;<br>Better prevention through early risk identification and management of acute care.	Personal health budgets;<br>Specific acute activities:<br>- Rapid assessment upon presentation to acute care<br>- Discharge support<br>- Visual ward model	Outcome

For peer review only - http://bmjopen.bmj.com/site/about/guidelines.xhtml

Greenwich	Older people with complex or chronic conditions; individuals with mental health issues	Integrated commissioning through joint health and social care assessment, planning and management;<br>Integrated care through multi-disciplinary teams including the voluntary sector, shared care plans, information and investment in self-care initiatives such as telehealth;<br>Better prevention and management of acute care.	Personal health budgets;<br>Specific acute activities:<br>- Risk stratification to identify 11.5% at highest risk of hospital admission within one year<br>- Virtual admission avoidance team<br>- Greenwich Joint Emergency Team (JET)<br>- Access to the medical diagnostic centre at the acute trust and outreach specialist opinion<br>- Hospital Intervention Discharge Team<br>- Community Assessment and Rehabilitation Teams (CARs).	Outcome
Islington	Whole population with focus on vulnerable older people;<br>people with long term conditions;<br>young people at risk;<br>people with mental health issues.	Integrated commissioning including patients through shared vision, planning, and information sharing;<br>Integrated care activities including information sharing;<br>Better prevention activities, including self-management support through personal health budgets and telehealth.	Incentivising acute and community healthcare services through CQUIN<br>Care pathways for conditions like COPD.	Aim
Kent	Adults with long term conditions and older people	Integrated commissioning through information sharing and data mapping;<br>Integrated care through multidisciplinary care teams organised around GP practices and workforce development;<br>Better prevention through risk identification, and self-management activities such as personal health budgets and telehealth.	Year of Care financial model and risk stratification;<br>Multidisciplinary care team meetings and neighbourhood care teams, integrated working within A&E departments, community integrated care centres;<br>Advanced Assistive technology partnership, joint working between paramedics and social care practitioners to respond quickly to 999 calls.	Aim

For peer review only - http://bmjopen.bmj.com/site/about/guidelines.xhtml

Leeds	Whole population	Integrated commissioning with shared vision, outcomes and budget;<br>Integrated care through health and social care teams focused around GP practice populations and workforce development;<br>Prevention through self-management activities such as telehealth.	Risk stratification and year of care model;<br>Divestment from emergency department; funding where appropriate to reinvest into community-based services;<br>Fully integrated health and social care bed unit	Outcome
North West London	Whole population	Integrated commissioning with a shared vision, responsibility, budget allocation and information sharing;<br>Integrated care through joined up services, incentives, multidisciplinary care teams and care plans;<br>Prevention and self-management through personalisation.	Integrated care organisation;<br>Bespoke IT tool to access patient data for both patients and care providers to help prevent readmission.	Aim
Southend	Whole population with focus on high service users	Integrated commissioning through a Pioneer Strategic Group, with a shared budget and information management;<br>Integrated care through increased involvement of the voluntary sector and patients in co-design and workforce development.	7-day multidisciplinary teams;<br>Extension of the Single Point of Referral (SPOR) to reduce avoidable admissions and delayed transfers of care;<br>Hospital discharge-step down scheme.	Outcome
South Devon and Torbay	Whole population	Integrated commissioning and governance arrangements with shared objectives, information sharing and bringing together leaders in the health and social care system;	Support for a 24/7 hospice at home service through a chosen care home provider, with a team of specialist nurses and senior healthcare assistants, a rapid response service and night driver team;<br>Pilot of Sunday working over three consecutive weekends on 5 wards;	Outcome

For peer review only

		Integrated care, not as an objective but an enabler, including an integrated care organisation providing acute, community and social care services 7 days a week, multidisciplinary teams including involvement of voluntary sector and workforce development;<br>Two programmes of work focused on better management of care and support for the elderly and young people.	Joined up IT including E-prescribing, E-booking and VitalPAC across healthcare organisations.	
South Tyneside	Whole population with focus on people who could benefit from initiatives on prevention, wellness promotion and self-care	Integrated commissioning and provision of care, through shared funding, joint decision-making, workforce development and information sharing.	Urgent care delivery group.<br>Predictive modelling to identify groups of patients vulnerable to hospital admission.	Aim
Stoke and North Staffordshire	Cancer and end-of-life care patients	Integrated service development through co-design by CCGs, Macmillan Cancer Support, Local Authorities and Public Health England to create accountable Service Integrator;<br>Integrated commissioning through shared arrangements across CCGs and social care and outcomes;<br>Integrated care through workforce development and co-design of care pathways.	Appointment of a principal provider for cancer and a principal provider for end of life care accountable for the determination of patient pathways through care.	-
Waltham Forest, East London and City	People at risk of hospital admission	Integrated commissioning through data sharing, joint	Whole System Demonstrator pilot.<br>Waltham Forest case management and rapid response programme.	Outcome

For peer review only

		health/social care assessment, joint contracting and payment; Integrated care through workforce development, restructuring of secondary care providers, greater involvement of the voluntary sector, rapid response and discharge tools, acute discharge support.		
Worcestershire	Whole population with focus on older people and people with long term conditions	Integrated commissioning through a shared funding of the oversight board, a shared vision, and community engagement.<br>Integrated care through service and clinical integration over organisational integration, reorganised acute care, multidisciplinary teams with involvement of the voluntary sector.<br>6 Transformation programme areas: Urgent Care; Out of hospital care; Specialised Commissioning; Acute Hospital Services; Future Lives; Children and Young People's Plan.	Virtual ward and integrated team projects.<br>Assistive technology for self-care and self-monitoring.<br>Improving Patient Flow strategy.<br>Personal Health Budgets.	Outcome

For peer review only

2. Local Authority to Pioneer Lookup

Local Authority Code	Local Authority	Pioneer
E08000016	Barnsley	Barnsley
E06000050	Cheshire West and Chester	Cheshire
E06000049	Cheshire East	Cheshire
E06000052	Cornwall	Cornwall and Isles of Scilly
E06000053	Isles of Scilly	Cornwall and Isles of Scilly
E09000011	Greenwich	Greenwich
E09000019	Islington	Islington
E07000105	Ashford	Kent
E07000106	Canterbury	Kent
E07000107	Dartford	Kent
E07000108	Dover	Kent
E07000109	Gravesham	Kent
E07000110	Maidstone	Kent
E07000111	Sevenoaks	Kent
E07000112	Shepway	Kent
E07000113	Swale	Kent
E07000114	Thanet	Kent
E07000115	Tonbridge and Malling	Kent
E07000116	Tunbridge Wells	Kent
E08000035	Leeds	Leeds
E09000005	Brent	North West London
E09000009	Ealing	North West London
E09000013	Hammersmith and Fulham	North West London
E09000015	Harrow	North West London
E09000017	Hillingdon	North West London
E09000018	Hounslow	North West London
E09000020	Kensington and Chelsea	North West London
E09000033	Westminster	North West London
E06000027	Torbay	South Devon and Torbay
E07000044	South Hams	South Devon and Torbay
E07000045	Teignbridge	South Devon and Torbay
E08000023	South Tyneside	South Tyneside
E06000033	Southend-on-Sea	Southend
E07000192	Cannock Chase	Stoke and North Staffordshire
E07000194	Lichfield	Stoke and North Staffordshire
E07000195	Newcastle-under-Lyme	Stoke and North Staffordshire
E07000197	Stafford	Stoke and North Staffordshire
E07000198	Staffordshire Moorlands	Stoke and North Staffordshire
E06000021	Stoke-on-Trent	Stoke and North Staffordshire
E07000196	South Staffordshire	Stoke and North Staffordshire
E09000031	Waltham Forest	Waltham Forest and East London and City
E09000025	Newham	Waltham Forest and East London and City
E09000030	Tower Hamlets	Waltham Forest and East London and City
E07000234	Bromsgrove	Worcestershire

For peer review only

E07000235	Malvern Hills	Worcestershire
E07000236	Redditch	Worcestershire
E07000237	Worcester	Worcestershire
E07000238	Wychavon	Worcestershire
E07000239	Wyre Forest	Worcestershire

For peer review only

For peer review only - http://bmjopen.bmj.com/site/about/guidelines.xhtml

3. Emergency admission rates for individual local authorities within Pioneers at baseline and follow up, with percentage differences compared to baseline

Local Authority Code	Local Authority	Emergency Admission Rate			Percentage Difference	
		Baseline	2014/15	2015/16	2014/15	2015/16
E08000016	Barnsley	10,992	11,769	12,325	7.07%	12.13%
E06000049	Cheshire East	11,485	12,537	12,954	9.17%	12.80%
E06000050	Cheshire West and Chester	11,033	11,782	11,963	6.79%	8.42%
E06000052/53	Cornwall and Isles of Scilly	8,170	8,061	8,193	-1.33%	0.29%
E09000011	Greenwich	8,168	8,226	9,513	0.71%	16.47%
E09000019	Islington	6,324	6,320	6,096	-0.06%	-3.60%
E07000105	Ashford	8,708	9,785	10,506	12.37%	20.65%
E07000106	Canterbury	9,663	10,503	10,499	8.70%	8.66%
E07000107	Dartford	10,325	12,037	11,063	16.59%	7.15%
E07000108	Dover	9,201	10,411	10,809	13.14%	17.47%
E07000109	Gravesham	9,529	10,825	10,379	13.60%	8.92%
E07000110	Maidstone	9,553	10,470	10,219	9.60%	6.97%
E07000111	Sevenoaks	8,420	8,842	8,114	5.01%	-3.64%
E07000112	Shepway	8,921	9,566	10,274	7.24%	15.17%
E07000113	Swale	10,590	10,746	12,117	1.47%	14.42%
E07000114	Thanet	9,245	9,952	10,194	7.65%	10.27%
E07000115	Tonbridge and Malling	8,842	8,632	8,448	-2.37%	-4.46%
E07000116	Tunbridge Wells	9,188	8,631	7,483	-6.06%	-18.55%
E08000035	Leeds	11,399	9,605	10,155	-15.74%	-10.91%
E09000005	Brent	8,882	8,541	8,425	-3.84%	-5.15%
E09000009	Ealing	10,246	9,828	10,398	-4.08%	1.49%
E09000013	Hammersmith and Fulham	10,312	9,588	9,876	-7.03%	-4.23%
E09000015	Harrow	8,397	8,430	8,084	0.39%	-3.73%
E09000017	Hillingdon	9,753	9,636	9,346	-1.20%	-4.18%
E09000018	Hounslow	8,998	9,751	11,053	8.36%	22.83%
E09000020	Kensington and Chelsea	7,688	7,094	7,048	-7.73%	-8.32%
E09000033	Westminster	7,095	6,454	6,266	-9.04%	-11.70%
E07000044	South Hams	6,591	6,413	6,962	-2.69%	5.63%
E07000045	Teignbridge	7,657	8,182	9,275	6.86%	21.13%
E06000027	Torbay	7,998	8,296	10,173	3.72%	27.19%
E08000023	South Tyneside	11,153	10,445	11,150	-6.35%	-0.03%
E06000033	Southend-on-Sea	9,243	10,397	10,224	12.49%	10.61%
E07000192	Cannock Chase	10,438	10,611	10,083	1.66%	-3.40%
E07000194	Lichfield	8,754	9,424	9,971	7.65%	13.90%
E07000195	Newcastle-under-Lyme	11,919	12,034	12,625	0.96%	5.92%
E07000196	South Staffordshire	8,353	8,907	9,575	6.64%	14.63%
E07000197	Stafford	10,289	10,441	9,900	1.47%	-3.78%
E07000198	Staffordshire Moorlands	8,448	8,649	9,162	2.37%	8.45%
E06000021	Stoke-on-Trent	11,438	11,704	12,959	2.32%	13.29%

For peer review only

E09000025	Newham	7,897	7,535	7,802	-4.58%	-1.20%
E09000030	Tower Hamlets	10,166	9,103	8,795	-10.45%	-13.49%
E09000031	Waltham Forest	9,489	9,333	8,242	-1.64%	-13.14%
E07000234	Bromsgrove	8,452	9,292	9,165	9.94%	8.43%
E07000235	Malvern Hills	8,289	7,937	7,360	-4.25%	-11.21%
E07000236	Redditch	10,556	10,650	11,534	0.89%	9.26%
E07000237	Worcester	9,858	9,180	9,705	-6.88%	-1.56%
E07000238	Wychavon	9,094	8,475	8,760	-6.81%	-3.68%
E07000239	Wyre Forest	7,855	7,366	7,511	-6.23%	-4.38%

4. Sensitivity analysis: excluding Stoke and North Staffordshire Pioneer

	2014/15	2015/16
**Model Coefficients** (p value)		
Intercept	-5.4279 (<0.0001)	-5.4297 (<0.0001)
Non-Pioneer/Pioneer	-0.0205 (0.3009)	-0.0204 (0.3023)
Baseline/Follow-Up	0.0487 (<0.0001)	0.0634 (<0.0001)
Interaction	-0.0302 (0.0489)	-0.0254 (0.1717)
**Percentage Difference** [95% confidence interval]		
Non-Pioneer	4.99 [3.81,6.18]	6.54 [5.21,7.89]
Pioneer	1.86 [-0.85,4.65]	3.87 [0.43,7.42]

Parallel trends interaction term p value = 0.3130

5. Sensitivity analysis: individual baseline years compared to 2014/15

**Baseline Year**	2010/11	2011/12	2012/13
**Parallel Trends P Value**	0.8272	0.3425	0.9235
**Model Coefficients** (p value)			
Intercept	-5.4173 (<0.0001)	-5.4524 (<0.0001)	-5.4144 (<0.0001)
Non-Pioneer/Pioneer	-0.0027 (0.9005)	-0.0034 (0.8652)	-0.0058 (0.7514)
Baseline/Time 2	0.0517 (<0.0001)	0.0522 (<0.0001)	0.0698 (<0.00001)
Interaction	-0.0290 (0.0916)	-0.0285 (0.0730)	-0.0258 (0.0195)
**Percentage Difference** [95% confidence interval]			
Non-Pioneer	5.30 [3.93,6.70]	5.36 [4.09,6.63]	7.23 [6.07,8.40]
Pioneer	2.29 [-0.95,5.64]	2.40 [0.46,5.34]	4.50 [2.48,6.55]

For peer review only - http://bmjopen.bmj.com/site/about/guidelines.xhtml

STROBE Statement—checklist of items that should be included in reports of observational studies

	Item No	Recommendation	Page No
**Title and abstract**	1	(*a*) Indicate the study's design with a commonly used term in the title or the abstract	1
		(*b*) Provide in the abstract an informative and balanced summary of what was done and what was found	2-3
**Introduction**			
Background/rationale	2	Explain the scientific background and rationale for the investigation being reported	4-5
Objectives	3	State specific objectives, including any prespecified hypotheses	5
**Methods**			
Study design	4	Present key elements of study design early in the paper	6
Setting	5	Describe the setting, locations, and relevant dates, including periods of recruitment, exposure, follow-up, and data collection	6-7
Participants	6	(*a*) *Cohort study*—Give the eligibility criteria, and the sources and methods of selection of participants. Describe methods of follow-up<br>*Case-control study*—Give the eligibility criteria, and the sources and methods of case ascertainment and control selection. Give the rationale for the choice of cases and controls<br>*Cross-sectional study*—Give the eligibility criteria, and the sources and methods of selection of participants	6-7
		(*b*) *Cohort study*—For matched studies, give matching criteria and number of exposed and unexposed<br>*Case-control study*—For matched studies, give matching criteria and the number of controls per case	-
Variables	7	Clearly define all outcomes, exposures, predictors, potential confounders, and effect modifiers. Give diagnostic criteria, if applicable	6-7
Data sources/ measurement	8*	For each variable of interest, give sources of data and details of methods of assessment (measurement). Describe comparability of assessment methods if there is more than one group	*6-7*
Bias	9	Describe any efforts to address potential sources of bias	8
Study size	10	Explain how the study size was arrived at	-
Quantitative variables	11	Explain how quantitative variables were handled in the analyses. If applicable, describe which groupings were chosen and why	7-8
Statistical methods	12	(*a*) Describe all statistical methods, including those used to control for confounding	7-8
		(*b*) Describe any methods used to examine subgroups and interactions	-
		(*c*) Explain how missing data were addressed	-
		(*d*) *Cohort study*—If applicable, explain how loss to follow-up was addressed<br>*Case-control study*—If applicable, explain how matching of cases and controls was addressed<br>*Cross-sectional study*—If applicable, describe analytical methods taking account of sampling strategy	-
		(*e*) Describe any sensitivity analyses	8

Continued on next page

For peer review only - http://bmjopen.bmj.com/site/about/guidelines.xhtml

**Results**			
Participants	13*	(a) Report numbers of individuals at each stage of study—eg numbers potentially eligible, examined for eligibility, confirmed eligible, included in the study, completing follow-up, and analysed	9
		(b) Give reasons for non-participation at each stage	-
		(c) Consider use of a flow diagram	-
Descriptive data	14*	(a) Give characteristics of study participants (eg demographic, clinical, social) and information on exposures and potential confounders	9
		(b) Indicate number of participants with missing data for each variable of interest	-
		(c) *Cohort study*—Summarise follow-up time (eg, average and total amount)	-
Outcome data	15*	*Cohort study*—Report numbers of outcome events or summary measures over time	-
		Case-control study—Report numbers in each exposure category, or summary measures of exposure	-
		Cross-sectional study—Report numbers of outcome events or summary measures	*9-10*
Main results	16	(*a*) Give unadjusted estimates and, if applicable, confounder-adjusted estimates and their precision (eg, 95% confidence interval). Make clear which confounders were adjusted for and why they were included	9-11
		(*b*) Report category boundaries when continuous variables were categorized	-
		(*c*) If relevant, consider translating estimates of relative risk into absolute risk for a meaningful time period	-
Other analyses	17	Report other analyses done—eg analyses of subgroups and interactions, and sensitivity analyses	-
**Discussion**			
Key results	18	Summarise key results with reference to study objectives	11
Limitations	19	Discuss limitations of the study, taking into account sources of potential bias or imprecision. Discuss both direction and magnitude of any potential bias	12-13
Interpretation	20	Give a cautious overall interpretation of results considering objectives, limitations, multiplicity of analyses, results from similar studies, and other relevant evidence	12-13
Generalisability	21	Discuss the generalisability (external validity) of the study results	12-14
**Other information**			
Funding	22	Give the source of funding and the role of the funders for the present study and, if applicable, for the original study on which the present article is based	15

*Give information separately for cases and controls in case-control studies and, if applicable, for exposed and unexposed groups in cohort and cross-sectional studies.

Note: An Explanation and Elaboration article discusses each checklist item and gives methodological background and published examples of transparent reporting. The STROBE checklist is best used in conjunction with this article (freely available on the Web sites of PLoS Medicine at http://www.plosmedicine.org/, Annals of Internal Medicine at http://www.annals.org/, and Epidemiology at http://www.epidem.com/). Information on the STROBE Initiative is available at www.strobe-statement.org.

For peer review only

BMJ Open

Area level impacts on emergency hospital admissions of the Integrated Care and Support Pioneer Programme in England: difference-in-differences analysis

Journal:	*BMJ Open*
Manuscript ID	bmjopen-2018-026509.R2
Article Type:	Research
Date Submitted by the Author:	12-Jul-2019
Complete List of Authors:	Keeble, Eilís; The Nuffield Trust<br>Bardsley, M; The Health Foundation<br>Durand, Mary Alison; London School of Hygiene and Tropical Medicine, Policy Innovation and Evaluation Research Unit, Department of Health Services Research and Policy<br>Hoomans, Ties ; London School of Hygiene and Tropical Medicine, Policy Innovation and Evaluation Research Unit, Department of Health Services Research and Policy<br>Mays, Nicholas; London School of Hygiene and Tropical Medicine, Policy Innovation and Evaluation Research Unit, Department of Health Services Research and Policy
**Primary Subject Heading**:	Health services research
Secondary Subject Heading:	Health policy
Keywords:	Quality in health care < HEALTH SERVICES ADMINISTRATION & MANAGEMENT, STATISTICS & RESEARCH METHODS, Organisation of health services < HEALTH SERVICES ADMINISTRATION & MANAGEMENT, Health policy < HEALTH SERVICES ADMINISTRATION & MANAGEMENT

SCHOLARONE™
Manuscripts

Area level impacts on emergency hospital admissions of the Integrated Care and Support Pioneer Programme in England: difference-in-differences analysis

Corresponding author: Eilís Keeble, The Nuffield Trust, 59 New Cavendish Street, London, W1G 7LP, United Kingdom, email: eilis.keeble@nuffieldtrust.org.uk, phone: 0207 631 8450

Martin Bardsley, The Health Foundation (Formerly of The Nuffield Trust), London, United Kingdom

Mary Alison Durand, Policy Innovation and Evaluation Research Unit, Department of Health Services Research and Policy, London School of Hygiene and Tropical Medicine, London, United Kingdom

Ties Hoomans, Policy Innovation and Evaluation Research Unit, Department of Health Services Research and Policy, London School of Hygiene and Tropical Medicine, London, United Kingdom

Nicholas Mays, Policy Innovation and Evaluation Research Unit, Department of Health Services Research and Policy, London School of Hygiene and Tropical Medicine, London, United Kingdom

Word Count: 3,248

For peer review only

ABSTRACT

Objective

To examine whether any differential change in emergency admissions could be attributed to integrated care by comparing Pioneer and non-Pioneer populations from a pre-Pioneer baseline period (April 2010 to March 2013) over two follow-up periods: to 2014/15 and to 2015/16.

Design

Difference-in-differences analysis of emergency hospital admissions from English Hospital Episode Statistics (HES).

Setting

Local authorities in England classified as either Pioneer or non-Pioneer.

Participants

Emergency admissions to all NHS hospitals in England with local authority determined by area of residence of the patient.

Intervention

Wave 1 of the Integrated Care and Support Pioneer Programme announced in November 2013.

Primary Outcome Measure

Change in hospital emergency admissions.

Results

The increase in the Pioneer emergency admission rate from baseline to 2014/15 was smaller at 1.98 per cent and significantly different from that of the non-Pioneers at 4.85 per cent (p=0.0395). The increase in the Pioneer emergency admission rate from baseline to 2015/16 was again smaller than for the non-Pioneers but the difference was not statistically significant (p=0.1905).

Conclusions

It is ambitious to expect unequivocal changes in a high level and indirect indicator of health and social care integration such as emergency hospital admissions to arise as a result of the

For peer review only

changes in local health and social care provision across organisations brought about by the Pioneers in their early years. We should treat any sign that the Pioneers have had such an impact with caution. Nevertheless, there does seem to be an indication from the current analysis that there were some changes in hospital use associated with the first year of Pioneer status that are worthy of further exploration.

STRENGTHS AND LIMITATIONS OF THIS STUDY

1. This study adds to the evidence of the impact of system-wide approaches to integrating health and social care, like the Integrated Care and Support Pioneer Programme, using advanced statistical methods to determine whether the Pioneers reduced emergency admissions.
2. Reducing emergency admissions is often cited as a key goal of new integrated models of care and the Hospital Episode Statistics provide a continuously collected person level dataset to enable tracking of changes over time at small area level.
3. Analysing the Pioneer sites collectively ensured the inclusion of a diverse range of areas which were unlikely to be systematically different at baseline from the non-Pioneers.
4. It is difficult to find a true counter-factual population to compare with the Pioneers as many other initiatives related to health and social care integration had been developed in other areas of the country previously and/or were being implemented almost simultaneously.
5. The Pioneers invested in a collection of health and social care integration strategies and interventions; identifying the causes and effects of these specific initiatives would require detailed local primary data collection but this analysis focuses on the overall impact of the Pioneers as a national policy initiative.

KEYWORDS

Organisation of health services, quality in health care, health policy, statistics and research methods

For peer review only

INTRODUCTION

In November 2013, the Integrated Care and Support Pioneer programme was initiated in England. The programme aimed to promote integration between the separate local health and social care systems in England by facilitating these systems to develop and implement new ways of working together with the objective of meeting people's needs better and improving service users' experience of care .[1]

In the first wave of the programme the English Department of Health (DH) (now Department of Health and Social Care (DHSC)) selected 14 Pioneer areas from a round of competitive applications, that were identified as the "most ambitious and visionary" in their plans for health and social care system integration.[2] Each Pioneer was given access to limited support and expertise over a five-year period and a one-off fund of £90,000 to help with initial development. A second wave of 11 Pioneer areas was subsequently announced in January 2015. These are excluded from the present analysis as there are insufficient time points available currently for an interpretable trend analysis.

Integration in the Pioneer areas has taken on different forms. As Erens and colleagues noted, *"What it meant to be 'a Pioneer' varied between sites and between individuals within sites. At various times it was apparent that Pioneer status meant one or more of the following:*

- *a 'badge' for a locality signifying national recognition of innovation and progress in integrating care*
- *an enabler of the existing local plan for transformation*
- *a particular governance arrangement, for example a Board that brought all system leaders and their organisations around the table*
- *a collection of discrete workstreams, characteristically covering a combination of different groups of users and infrastructure projects (for example, information sharing, workforce development, etc.)*
- *a specific new integrated service, such as a frailty service*
- *an ethos or way of thinking about and providing care, rather than a specific plan or set of initiatives"*[3]

For peer review only

Some of the Pioneers planned to focus on specific populations. Of these, the most common were older people, people with long term conditions and people at high risk of hospitalisation. Broadly, however, the Pioneers shared the same vision for the future of the health and social care system by seeking to create a ‘whole system’ of integrated care involving all local bodies and professional groups organised around the needs of individuals and their informal carers which set them apart from the rest of England.[4]

All but one (Stoke and North Staffordshire) of the Wave 1 Pioneers stated that reducing emergency admissions was an aim or an expected outcome of integration in their original bid. Risk stratification with targeted interventions and introducing preventive strategies to avoid the need for acute hospitalisation were listed as activities to achieve this goal (see supplementary material). The focus on reducing emergency hospital care use was given still greater emphasis by the Pioneers as financial austerity bit more deeply into local health care budgets after 2013.[3]

As a consequence of the focus on emergency hospital care as a costly service, the success of integrated care initiatives has often been presented, at least in part, in terms of their ability to reduce the need for emergency hospital admissions and to reduce emergency admission rates.[5] Reducing emergency admission rates has been a feature of English health policy over the past decade and continues to be one of the most commonly used measures of success for system change initiatives.[6–8] To date, however, there has been little evidence of initiatives successfully reducing emergency admissions.[9–11]

This paper presents new evidence on the effect of the Pioneer programme on emergency admissions. We investigate changes in the emergency admissions to hospitals of patients across England following the implementation of the programme in 2013. The analysis is part of a wider programme of evaluation of the Pioneers (http://piru.lshtm.ac.uk/projects/current-projects/integrated-care-pioneers-evaluation.html). Though it is not possible to identify precisely which elements of the programme, if any, led to any differential change observed (since the Pioneers were not working from an agreed template), such an analysis can be justified as a necessary step in understanding the impacts of a major initiative such as the Integrated Care and Support Pioneer programme, especially since it had much in common with successive initiatives such

For peer review only

as the New Care Model Vanguards and the current focus on Integrated Care Systems (ICSs).[12-13] The underlying hypothesis is that the cumulative effect of the specific initiatives embedded in each Pioneer programme would bring about sufficient change in emergency hospital care use as to be detectable at the level of the whole population of the Pioneers.

METHODS

To examine whether differential change in emergency admissions could be attributed to Pioneer status we used a difference-in-differences approach. Difference-in-differences measures the effect of the intervention (the Pioneer programme) by looking at the change in emergency admissions between the pre- and post- intervention periods in the two groups and quantifies whether or not the population within the Pioneer programme experiences a change that is significantly different to the comparison group, the non-Pioneers.

Data Sources

We used inpatient Hospital Episode Statistics (HES) to identify all emergency admissions to NHS hospitals in Pioneer and non-Pioneer areas across England. HES is collated by NHS Digital and is a pseudonymous patient level dataset that records basic features of admissions to hospital including: patient age, sex, admission date and an emergency admission indicator (admission methods starting with "2").[14]

To be able to compare emergency admission rates between areas (Pioneer/non-Pioneer), we also obtained information on key local authority level factors determining local population health and care needs:

- Demographic composition (age and sex), from the Office for National Statistics (ONS).[15]
- Deprivation decile, from the 2015 Index of Multiple Deprivation.[16]

Defining Pioneer Areas

The Pioneer areas did not all map neatly to a single set of health or local government administrative boundaries. After consultation with each Pioneer, they were mapped to the local authorities which most closely aligned with the intervention area (see supplementary material for lookup table). Local authority boundaries were used instead of health boundaries as the population denominators could be linked over a longer period. A wider breadth of data

For peer review only

is available for this boundary which is being used in other parts of the evaluation, for example social care data.

The local authorities which were linked to the second wave of Pioneers, initiated in January 2015, were excluded from all analyses and not included in either the Pioneer or non-Pioneer populations. Non-Pioneer areas were defined as any local authority that was not a first or second wave Pioneer.

Defining Time Periods

A baseline period before Pioneer programme implementation of April 2010 to March 2013 was compared to two follow-up periods: April 2014 to March 2015 (2014/15) and April 2015 to March 2016 (2015/16). The period April 2013 to March 2014 was excluded as this encompassed the call for applications to the programme (May 2013) and the announcement of the sites (November 2013).

Outcome

Our primary outcome was the average percentage difference in rates of emergency hospital admissions per 100,000 between baseline and follow-up (2014/15 or 2015/16) for the study groups (Pioneers/non-Pioneers). Area-level rates were calculated as the total number of emergency admissions over each time period divided by the mid-year population for each group. Admissions were derived by month and local authority of residence. They were adjusted for deprivation decile, age group (0-19, 20-39, 40-59, 60-79, 80+) and sex. The English age, sex and deprivation decile structure was used as the reference population for each local authority for the initial analysis. The secondary outcome was the difference in average percentage change in the rates over time between the Pioneers and non-Pioneers.

Statistical Analyses

An initial difference-in-differences comparison was performed by looking at the change in the adjusted emergency admission rate for the Pioneers and non-Pioneers. Percentage differences between the baseline period and the two follow-up time points of 2014/15 and 2015/16 were calculated, along with the difference between these.

To determine whether the change in emergency admissions in the Pioneers was significantly different from the change in the non-Pioneers, we performed difference-in-differences

For peer review only

regression analysis. We estimated negative binomial regression models for count data adjusting for age, sex and deprivation decile. Poisson models were first attempted but the data were over-dispersed and unsuitable. Each regression model included a continuous local authority population size exposure variable, a binary Pioneer status term (Pioneer/non-Pioneer), a binary time term (baseline/follow-up), a difference-in-differences term (Pioneer status*time) and covariate terms. We obtained robust standard error estimates adjusting for clustering of the repeated measures from each local authority. Significance was assessed at $p<0.05$. SAS 9.4® was used for all analyses.

For peer review only

Difference-in-Differences Estimation Validation Tests

To validate our difference-in-difference estimations, we tested the following assumptions:

1. That areas were not selected into the Programme based on emergency admission rates at baseline, by comparing baseline emergency admissions and demographics of the Pioneer and non-Pioneers.
2. That changes in emergency admission rates over time would be the same for both the Pioneer and non-Pioneer areas in the absence of the Pioneer programme, by comparing adjusted emergency admission rates for the Pioneers and non-Pioneers over the baseline period. These were compared graphically and statistically using a linear time trend of month in the baseline period interacted with Pioneer status controlling for age, sex and deprivation decile.

Sensitivity Analyses

We examined sensitivity of the main findings to excluding Stoke and North Staffordshire Pioneer from our analyses and to using individual years for the baseline period (see supplementary material). Stoke and North Staffordshire had a unique target population and no focus on reducing emergency admissions. As the baseline period covered three years, each individual baseline year was also compared to the first follow-up time point.

Patient and Public Involvement

Patient and public representatives are involved in the wider evaluation of which this analysis forms a part and were involved in the selection and peer review of the initial proposal on which this analysis is based.

RESULTS

Baseline Characteristics

The characteristics of the Pioneers and non-Pioneers during the baseline period of April 2010 to March 2013 are summarised in Table 1. The Pioneers consisted of 49 local authorities and encompassed 17 percent of the English population in the baseline period.[15] The proportions of the population aged 65 and over, or female, were similar between the two groups. Area level deprivation in the Pioneers was slightly higher than in the non-Pioneers.

Table 1 Baseline Characteristics of the Pioneer and non-Pioneer populations

**Characteristic**	**Pioneers First Wave (n = 14)***	**Non-Pioneers**
Number of Local Authorities	49	244
Average Yearly Population at Baseline	9,083,051	37,137,613
Proportion Population Under 20	24%	24%
Proportion Population Aged 65+	16%	17%
Proportion Population Female	50%	50%
Average Local Authority IMD Score (2015)	21.1	18.7

* 11 second wave Pioneers and 33 associated local authorities were excluded from the analyses

Trend Analysis

Figure 1 shows the adjusted monthly emergency admission rates for the Pioneers and non-Pioneers between April 2010 and March 2016. On visual inspection, the trends in the baseline period overlap which indicates that trend bias should have limited impact on the difference-in-differences analysis. A statistical test of the trends in the baseline period also indicated limited trend bias (p=0.7378).

Difference-in-Differences

Between the baseline period and the first follow-up period (2014/15) average emergency admission rates decreased by 0.42% for the Pioneers and increased by 3.46% for the non-Pioneers, with a difference-in-differences of 3.89% (see Table 2). When the baseline was compared to the second follow-up period (2015/16), the Pioneers still had a lower increase at 2.23% but the difference compared to the non-Pioneers was smaller at 3.23%.

For peer review only

Table 2 Emergency admission rates for Pioneers and Non-Pioneers (adjusted for age, sex and deprivation decile) at baseline and follow-up, with percentage differences compared to baseline and difference-in-differences between non-Pioneers and Pioneers

	Emergency Admission Rate (per 100,000 population)			Percentage Difference		Difference-in-Differences*	
	Baseline	2014/15	2015/16	2014/15	2015/16	2014/15	2015/16
Non-Pioneer	9,942	10,287	10,485	3.46%	5.46%	3.89%	3.23%
Pioneer	9,948	9,906	10,170	-0.42%	2.23%		

** Difference between the non-Pioneer and Pioneer percentage differences, positive value indicates non-Pioneer change is greater*

Trends for the individual Pioneers varied. For example, half the Pioneers had a percentage increase in their emergency admission rates between baseline and 2014/15, while the percentage difference for the Pioneers as a whole was a slight decrease (see Table 3). There was also variation within Pioneers (see supplementary material). For example, the constituent local authorities comprising the Waltham Forest, East London and City Pioneer had declines in emergency admission rates ranging from -10.45% (Tower Hamlets) to -1.64% (Newham) between baseline and 2014/15, while the overall percentage difference was -5.73%.

Table 3 Emergency admission rates for individual Pioneers (adjusted for age, sex and deprivation decile) at baseline and follow-up, with percentage differences compared to baseline

Pioneer (Number of LAs)	Emergency Admission Rate (per 100,000 population)			Percentage Difference to Baseline	
	Baseline	2014/15	2015/16	2014/15	2015/16
Barnsley (1)	10,992	11,769	12,325	7.07%	12.13%
Cheshire (2)	11,259	12,160	12,459	8.00%	10.65%
Cornwall and Isles of Scilly (2)	8,170	8,061	8,193	-1.33%	0.29%
Greenwich (1)	8,168	8,226	9,513	0.71%	16.47%
Islington (1)	6,324	6,320	6,096	-0.06%	-3.60%
Kent (12)	9,349	10,033	10,009	7.32%	7.06%
Leeds (1)	11,399	9,605	10,155	-15.74%	-10.91%
North West London (8)	8,922	8,665	8,812	-2.87%	-1.23%
South Devon and Torbay (3)	7,415	7,630	8,803	2.90%	18.72%
South Tyneside (1)	11,153	10,445	11,150	-6.35%	-0.03%
Southend (1)	9,243	10,397	10,224	12.49%	10.61%
Stoke and North Staffordshire (7)	9,949	10,253	10,611	3.06%	6.66%
Waltham Forest, East London and City (3)	9,184	8,657	8,279	-5.73%	-9.85%
Worcestershire (6)	9,018	8,817	9,006	-2.23%	-0.13%

For peer review only

Difference-in-Differences Regression

After adjusting for age, sex and deprivation, the difference-in-differences regression analysis showed that the change in emergency admission rates in the Pioneers between baseline and 2014/15 was smaller and significantly different from that of the non-Pioneers (p=0.0394) (see Table 4). The Pioneer emergency admission rate increased by 1.98 per cent compared to 4.85 per cent in the non-Pioneers. When comparing baseline with 2015/16, the analysis still indicated that the change in emergency admissions for the Pioneers was smaller at 4.07 per cent compared to 6.36 percent for the non-Pioneers but the difference was not statistically significant (p=0.1905).

Table 4 Difference in difference model coefficients and percentage difference in emergency admissions for Pioneers and Non-Pioneers, adjusted for age, sex and deprivation

	2014/15	2015/16
**Model Coefficients** (p value)		
Intercept	-5.4234 (<0.0001)	-5.4259 (<0.0001)
Non-Pioneer/Pioneer	-0.0044 (0.8169)	-0.0043 (0.8190)
Baseline/Follow-Up	0.04173 (<0.0001)	0.0617 (<0.0001)
Interaction	-0.0278 (0.0394)	-0.0218 (0.1905)
**Percentage Difference** [95% confidence interval]		
Non-Pioneer	4.85 [3.68,6.03]	6.36 [5.05,7.70]
Pioneer	1.98 [-0.37,4.38]	4.07 [1.03,7.20]

Sensitivity Analyses

Excluding Stoke and North Staffordshire did not affect the overall findings but reduced the significance of the difference between the Pioneers and non-Pioneers in 2014/15 (p=0.0489), however, this exclusion also meant the trends were less parallel and subject to more bias from the baseline period (p=0.3130). After comparing individual baseline years to 2014/15, all years found a smaller change for the non-Pioneers but only 2012/13 was statistically significant (p=0.0195), this was also the baseline year with the most parallel trends for Pioneers and non-Pioneers (p=0.9235). Full results presented in supplementary material.

DISCUSSION

The Integrated Care and Support Pioneers represent one important example of how English health and social care services have been exploring new ways of working across organisational boundaries. The aims of the individual Pioneers varied,[3] but most had a common interest in

For peer review only

providing care and support that was intended to reduce the need for urgent care services and lead to a reduction in emergency hospital admissions. After comparing changes in emergency admissions from a three-year pre-Pioneer baseline period between Pioneer and non-Pioneer populations, we found a lower increase in emergency admissions for the Pioneers than the non-Pioneers. This lower increase was statistically significant for the comparison between baseline and 2014/15 (p=0.0394) but not for the comparison between baseline and 2015/16 (p=0.1905).

This type of population level analysis can help provide some independent evidence of the likely scale of changes within an area associated with integrated care initiatives and curb some of the more zealous rhetoric for or against integrated health and social care, and the related changes in service delivery. Looking at emergency admission data on this scale means the outcome of interest is based on a relatively large number of events and continuously collected data – making them useful as a measure of potential programme impact. This is in contrast to a range of other potential measures of health and social care integration at community level that are likely to be less sensitive to short term change such as annual patient experience surveys. The size and range of geographical areas covered by both the Pioneers and non-Pioneers along with their socio-demographic similarities should mean that differences in factors such as supply of social care services or acute hospital beds and the process of collecting data are unlikely to be systematically different between the two groups beyond any changes associated with Pioneer status.

It would be beneficial to track emergency admissions for more than two years to measure the impact of policy initiatives such as the Pioneers more definitively. However, during the life of the Pioneer programme, there were parallel changes in the wider policy context both in terms of specific health and care integration policies such as the Better Care Fund,[17] the overall level of funding for both health and social care in a period of unprecedented financial austerity and,[18] from 2015 onwards, the New Care Model Vanguards.[19] In particular, the Vanguards' approach to improving care coordination had much in common with the Pioneers. This means that the ideas behind integration that prompted the Pioneers and the types of interventions that they developed are no longer (if they ever were) unique to these areas and are being implemented across the country. Therefore, a true counter-factual population is difficult to find. This may, in part, explain why the difference between the Pioneers and non-

For peer review only

Pioneers reduced between baseline and 2015/16 compared with baseline and 2014/15 as the behaviour of the non-Pioneers becomes increasingly similar to the Pioneers.[20] This is in part to be expected as disseminating learning from the Pioneers was actively encouraged as part of the programme.

In addition to the difficulties of finding a counterfactual over the life-time of the Pioneers, it was not the first programme to focus on health and social care integration in England. One such previous initiative was the Integrated Care Pilots. While an effect on emergency admissions was not found for this programme, it can't be ruled out that these pilots have had a legacy impact on emergency admissions.[21] It should therefore be noted that, three of the Pioneers overlap with areas that were previously Integrated Care Pilots (Cornwall, Torbay and Tower Hamlets) and therefore, may have had a focus on integration for longer than some other Pioneers. This may in part explain the steady declines in emergency admissions seen in Tower Hamlets and to a lesser extent Cornwall. Seven of the Integrated Care Pilots also covered areas which were not Pioneers and therefore, the impact of the Pioneers in contrast to these may be reduced.

A more detailed understanding of the impacts of the Pioneers would be gained with a targeted analytical approach using information on the specific initiatives implemented in each Pioneer and data on the exact populations in receipt of these initiatives (this is being attempted in another component of the Pioneer evaluation). While this might yield gains in terms of causal inference in that changes could potentially be attributed to a specific set of local actions, such an analysis might lose the ability to assess the impact of change across a system and an entire population. This is important to note as the Pioneers were intended to be a complex mix of specific service changes and initiatives, supported by a wider pattern of infrastructural changes at the level of the local health and social care system.

Other studies have looked at schemes with an aspiration to reduce the need for urgent hospital care through better coordinated health and care services, and with an emphasis on preventing admissions. Success is typically assessed in terms of reduction in emergency hospital admissions and various previous evaluations show that this has been difficult to achieve.[9–11] Despite the intense policy interest in how different forms of service delivery can reduce emergency admissions, there are few, if any, studies showing unequivocal change

For peer review only

in the direction desired. Against this backdrop, the modest changes observed across the 14 wave one Pioneer areas in their first two years look promising. However, when exploring the extent to which the observed changes are likely to be related causally to Pioneer status, it should be noted that:

a. the effect appears to be temporary: and as such the effect may have been linked to changes that took place in the early stages of the Pioneers or pre-Pioneer but were not sustained; or the non-Pioneer areas introduced changes which have subsequently reduced the difference between them and the Pioneers; and
b. the changes in emergency admissions were not shown in all places and even varied between local authority areas within the same Pioneer.

Conclusion

It is ambitious to expect unequivocal changes in a single high level and indirect indicator of health and care integration such as emergency hospital admissions to arise as a result of changes in local health and care provision across organisations brought about by the Pioneers in their early years. We should treat any signs that the Pioneers have had such an impact with caution. Nevertheless, our analysis does seem to provide some evidence that there were some changes in hospital use associated with the first year of Pioneer status that are worthy of further exploration. At the very least, this analysis shows that Pioneer status does not seem to have been associated with a relative deterioration in performance in terms of emergency hospital use.

FIGURE LEGEND

Figure 1 Emergency admission rate for Pioneers and Non-Pioneers by month adjusted for age, sex and deprivation decile (Pioneer intervention introduced in shaded area)

AUTHOR CONTRIBUTIONS

EK undertook the analysis and with MB drafted the initial paper. MB and NM contributed to design of the study. EK, MB, MAD, TH and NM contributed to interpretation of findings and revisions of the paper.

For peer review only

ACKNOWLEDGEMENTS

This work uses data provided by patients and collected by the NHS as part of their care and support. Read more at https://www.nuffieldtrust.org.uk/about/corporate-policies#information-security-and-data.

Hospital Episode Statistics data (year range 2007/08–2015/16) Copyright © (2018), NHS Digital. Re-used with the permission of NHS Digital. All rights reserved.

FUNDING

This study is funded by the NIHR Policy Research Programme (*Evaluation of the Integrated Care and Support Pioneers Programme in the context of new funding arrangements for integrated care in England* (2015-2020), PR-R10-0514-25001). The views expressed are those of the authors and are not necessarily those of the NIHR or the Department of Health and Social Care.

COMPETING INTERESTS

This study is funded by the NIHR Policy Research Programme (*Evaluation of the Integrated Care and Support Pioneers Programme in the context of new funding arrangements for integrated care in England* (2015-2020), PR-R10-0514-25001). The views expressed are those of the authors and are not necessarily those of the NIHR or the Department of Health and Social Care.

DATA AVAILABILITY

This study used Hospital Episode Statistics data obtained from NHS Digital under a data sharing agreement and are reused with their permission. Hospital Episode Statistics data may be obtained from NHS Digital under a similar process but we are unable to share it per the terms of our agreement.

For peer review only

REFERENCES

[1] NHS England. Integrated care pioneers announced. 2013. Available: https://www.england.nhs.uk/2013/11/interg-care-pioneers/. [Accessed: 01-Aug-2018].

[2] Department of Health. Letter inviting expressions of interest for health and social care integration "Pioneers". 2013. Available: https://www.gov.uk/government/publications/social-care-integration-pioneers. [Accessed: 01-Aug-2018].

[3] Erens B, Wistow G, Mounier-Jack S, et al. Early evaluation of Integrated Care and Support Pioneers Programme: Final Report. Policy Innovation Research Unit. 2015. Available: http://piru.lshtm.ac.uk/assets/files/Early_evaluation_of_IC_Pioneers_Final_Report.pdf . [Accessed: 01-Aug-2018].

[4] National Collaboration for Integrated Care and Support. Integrated Care and Support: Our Shared Commitment. 2013. Available: https://assets.publishing.service.gov.uk/government/uploads/system/uploads/attachment_data/file/198748/DEFINITIVE_FINAL_VERSION_Integrated_Care_and_Support_-_Our_Shared_Commitment_2013-05-13.pdf. [Accessed: 01-Aug-2018].

[5] Raleigh V, Bardsley M, Smith P, et al. Integrated care and support Pioneers: Indicators for measuring the quality of integrated care: Final report. Policy Innovation Research Unit. 2014. Available: http://piru.lshtm.ac.uk/assets/files/IC_and_support_Pioneers-Indicators.pdf. [Accessed: 01-Aug-2018].

[6] National Audit Office. Emergency admissions to hospital: managing the demand. National Audit Office. 2013. Available: https://www.nao.org.uk/wp-content/uploads/2013/10/10288-001-Emergency-admissions.pdf. [Accessed: 01-Aug-2018].

[7] Wallace E, Smith SM, Fahey T, et al. Reducing emergency admissions through community based interventions. *BMJ* 2016;352:h6817 doi:10.1136/bmj.h6817 [published Online first: 28 January 2016].

[8] Lloyd T, Wolters A, Steventon A. The impact of providing enhanced support for care home residents in Rushcliffe. The Health Foundation. 2017. Available: https://www.health.org.uk/publication/impact-enhanced-support-rushcliffe. [Accessed: 01-Aug-2018].

For peer review only

[9] Bardsley M, Steventon A, Smith J, et al. Evaluating integrated and community-based care: How do we know what works? The Nuffield Trust. 2013. Available: https://www.nuffieldtrust.org.uk/files/2017-01/evaluating-integrated-community-care-web-final.pdf. [Accessed: 01-Aug-2018].

[10] Roland M, Lewis R, Steventon A, et al. Case management for at-risk elderly patients in the English integrated care pilots: observational study of staff and patient experience and secondary care utilisation. *Int J Integr Care* 2012;12(5) http://doi.org/10.5334/ijic.850.

[11] Purdy S, Paranjothy S, Huntley A, et al. Interventions to reduce unplanned hospital admission: a series of systematic reviews. National Institute for Health Research. 2012. Available: http://www.bristol.ac.uk/media-library/sites/primaryhealthcare/migrated/documents/unplannedadmissions.pdf. [Accessed: 01-Aug-2018].

[12] NHS England. New Care Models: Vanguards – developing a blueprint for the future of the NHS and care services. 2016. Available: https://www.england.nhs.uk/wp-content/uploads/2015/11/new_care_models.pdf [Accessed: 11-Jun-2019]

[13] NHS England. Breaking down barriers to better health and care. 2019. Available: https://www.england.nhs.uk/wp-content/uploads/2019/04/breaking-down-barriers-to-better-health-and-care-march19.pdf [Accessed: 11-Jun-2019]

[14] NHS Digital. Hospital Episode Statistics data 2007/08-2015/16.

[15] Office for National Statistics. Population estimates 2007-2015. Available: https://www.ons.gov.uk/peoplepopulationandcommunity/populationandmigration/populationestimates. [Accessed: 14-May-2018].

[16] Ministry of Housing, Communities and Local Government. English indices of deprivation 2015. Available: https://www.gov.uk/government/statistics/english-indices-of-deprivation-2015. [Accessed: 12-Jun-2018].

[17] NHS England. Better Care Fund. Available: https://www.england.nhs.uk/ourwork/part-rel/transformation-fund/bcf-plan/. [Accessed: 14-May-2018].

[18] Robertson R, Wenzel L, Thompson J, et al. Understanding NHS financial pressures: How are they affecting patient care? The Kings Fund. 2017.

For peer review only

[19] NHS. NHS Five Year Forward View. 2014. Available: https://www.england.nhs.uk/wp-content/uploads/2014/10/5yfv-web.pdf. [Accessed: 01-Aug-2018].

[20] Chen YF, Hemming K, Stevens AJ, et al. Secular trends and evaluation of complex interventions: the rising tide phenomenon. *BMJ Qual Saf 2016;*25(5)303–310 doi: 10.1136/bmjqs-2015-004372 [published Online first: 06 Oct 2015].

[21] RAND Europe and Ernst & Young LLP. National Evaluation of the Department of Health's Integrated Care Pilots: Final Report. 2012. Available: https://assets.publishing.service.gov.uk/government/uploads/system/uploads/attachment_data/file/215103/dh_133127.pdf [Accessed: 14-Jun-2019]

For peer review only

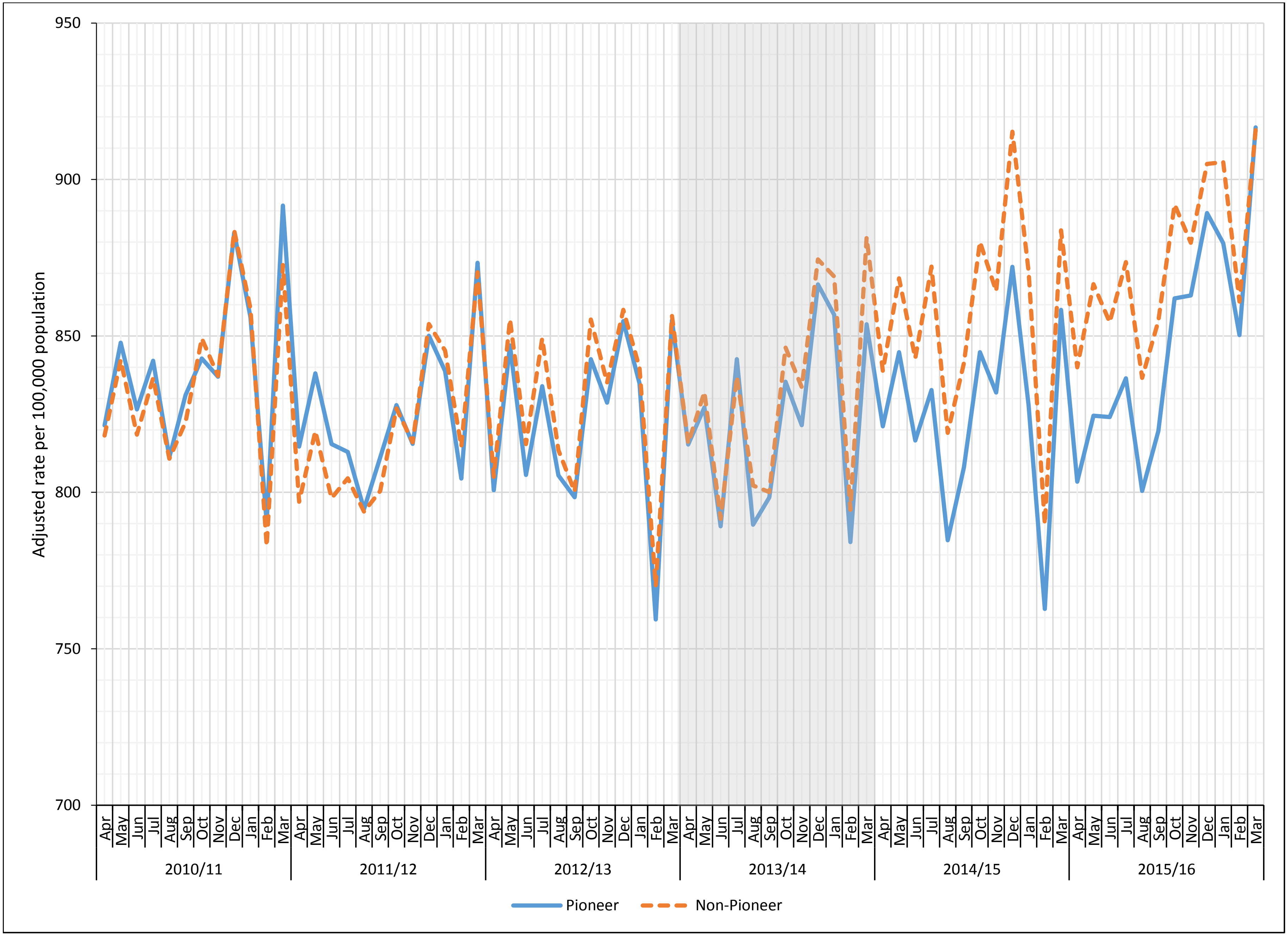

For peer review only - http://bmjopen.bmj.com/site/about/guidelines.xhtml

SUPPLEMENTARY MATERIAL

1. Target populations, integration mechanisms, activities and emergency admission focus of Wave 1 Pioneers
2. Local Authority to Pioneer Lookup
3. Emergency admission rates for individual local authorities within Pioneers
4. Sensitivity analysis: excluding Stoke and North Staffordshire Pioneer
5. Sensitivity analysis: individual baseline years compared to 2014/15

For peer review only

1. Target populations, integration mechanisms, activities and emergency admission focus of Wave 1 Pioneers

Pioneer	Target Population(s)	Integration Mechanisms	Examples of Specific Activities	Reduced emergency admissions
Barnsley	Whole population with focus on children and families	Integrated programme boards;<br>Adopting a citizenship approach at all levels of the community to move from intervention to prevention;<br>Provision of information, advice, and signposting through services such as telecare.	Universal information and advice service;<br>Be Well Barnsley: community orientated prevention;<br>Immediate care review;<br>Integrated personal budgets	Aim
Cheshire	Older adults with chronic conditions; individuals with mental health issues; complex needs families	Integration commissioning through a joint governance board, redesigned care and intervention pathways and joint investment plan involving the voluntary and community sectors;<br>Integration of care management and investment into integration enablers, such as shared records and telehealth services.	New funding/contracting model for the acute sector and community care;<br>Hospital at home;<br>Partnership working between Health Care Management Financial Association, Health Care Services and Net Orange to reduce hospital admissions;	Outcome
Cornwall and Isles of Scilly	People who are at risk of becoming high users of health and social care services	Integrated leadership through shared goals and performance measures, information sharing and a new funding contract;<br>Investment in telehealth;<br>Integrated care through multi-disciplinary teams, workforce development, information sharing;<br>Better prevention through early risk identification and management of acute care.	Personal health budgets;<br>Specific acute activities:<br>- Rapid assessment upon presentation to acute care<br>- Discharge support<br>- Visual ward model	Outcome

Greenwich	Older people with complex or chronic conditions; individuals with mental health issues	Integrated commissioning through joint health and social care assessment, planning and management;<br>Integrated care through multi-disciplinary teams including the voluntary sector, shared care plans, information and investment in self-care initiatives such as telehealth;<br>Better prevention and management of acute care.	Personal health budgets;<br>Specific acute activities:<br>- Risk stratification to identify 11.5% at highest risk of hospital admission within one year<br>- Virtual admission avoidance team<br>- Greenwich Joint Emergency Team (JET)<br>- Access to the medical diagnostic centre at the acute trust and outreach specialist opinion<br>- Hospital Intervention Discharge Team<br>- Community Assessment and Rehabilitation Teams (CARs).	Outcome
Islington	Whole population with focus on vulnerable older people;<br>people with long term conditions;<br>young people at risk;<br>people with mental health issues.	Integrated commissioning including patients through shared vision, planning, and information sharing;<br>Integrated care activities including information sharing;<br>Better prevention activities, including self-management support through personal health budgets and telehealth.	Incentivising acute and community healthcare services through CQUIN<br>Care pathways for conditions like COPD.	Aim
Kent	Adults with long term conditions and older people	Integrated commissioning through information sharing and data mapping;<br>Integrated care through multidisciplinary care teams organised around GP practices and workforce development;<br>Better prevention through risk identification, and self-management activities such as personal health budgets and telehealth.	Year of Care financial model and risk stratification;<br>Multidisciplinary care team meetings and neighbourhood care teams, integrated working within A&E departments, community integrated care centres;<br>Advanced Assistive technology partnership, joint working between paramedics and social care practitioners to respond quickly to 999 calls.	Aim

For peer review only

Leeds	Whole population	Integrated commissioning with shared vision, outcomes and budget;<br>Integrated care through health and social care teams focused around GP practice populations and workforce development;<br>Prevention through self-management activities such as telehealth.	Risk stratification and year of care model;<br>Divestment from emergency department; funding where appropriate to reinvest into community-based services;<br>Fully integrated health and social care bed unit	Outcome
North West London	Whole population	Integrated commissioning with a shared vision, responsibility, budget allocation and information sharing;<br>Integrated care through joined up services, incentives, multidisciplinary care teams and care plans;<br>Prevention and self-management through personalisation.	Integrated care organisation;<br>Bespoke IT tool to access patient data for both patients and care providers to help prevent readmission.	Aim
Southend	Whole population with focus on high service users	Integrated commissioning through a Pioneer Strategic Group, with a shared budget and information management;<br>Integrated care through increased involvement of the voluntary sector and patients in co-design and workforce development.	7-day multidisciplinary teams;<br>Extension of the Single Point of Referral (SPOR) to reduce avoidable admissions and delayed transfers of care;<br>Hospital discharge-step down scheme.	Outcome
South Devon and Torbay	Whole population	Integrated commissioning and governance arrangements with shared objectives, information sharing and bringing together leaders in the health and social care system;	Support for a 24/7 hospice at home service through a chosen care home provider, with a team of specialist nurses and senior healthcare assistants, a rapid response service and night driver team;<br>Pilot of Sunday working over three consecutive weekends on 5 wards;	Outcome

For peer review only - http://bmjopen.bmj.com/site/about/guidelines.xhtml

		Integrated care, not as an objective but an enabler, including an integrated care organisation providing acute, community and social care services 7 days a week, multidisciplinary teams including involvement of voluntary sector and workforce development;<br>Two programmes of work focused on better management of care and support for the elderly and young people.	Joined up IT including E-prescribing, E-booking and VitalPAC across healthcare organisations.	
South Tyneside	Whole population with focus on people who could benefit from initiatives on prevention, wellness promotion and self-care	Integrated commissioning and provision of care, through shared funding, joint decision-making, workforce development and information sharing.	Urgent care delivery group.<br>Predictive modelling to identify groups of patients vulnerable to hospital admission.	Aim
Stoke and North Staffordshire	Cancer and end-of-life care patients	Integrated service development through co-design by CCGs, Macmillan Cancer Support, Local Authorities and Public Health England to create accountable Service Integrator;<br>Integrated commissioning through shared arrangements across CCGs and social care and outcomes;<br>Integrated care through workforce development and co-design of care pathways.	Appointment of a principal provider for cancer and a principal provider for end of life care accountable for the determination of patient pathways through care.	-
Waltham Forest, East London and City	People at risk of hospital admission	Integrated commissioning through data sharing, joint	Whole System Demonstrator pilot.<br>Waltham Forest case management and rapid response programme.	Outcome

For peer review only

		health/social care assessment, joint contracting and payment; Integrated care through workforce development, restructuring of secondary care providers, greater involvement of the voluntary sector, rapid response and discharge tools, acute discharge support.		
Worcestershire	Whole population with focus on older people and people with long term conditions	Integrated commissioning through a shared funding of the oversight board, a shared vision, and community engagement.<br>Integrated care through service and clinical integration over organisational integration, reorganised acute care, multidisciplinary teams with involvement of the voluntary sector.<br>6 Transformation programme areas: Urgent Care; Out of hospital care; Specialised Commissioning; Acute Hospital Services; Future Lives; Children and Young People's Plan.	Virtual ward and integrated team projects.<br>Assistive technology for self-care and self-monitoring.<br>Improving Patient Flow strategy.<br>Personal Health Budgets.	Outcome

For peer review only

2. Local Authority to Pioneer Lookup

Local Authority Code	Local Authority	Pioneer
E08000016	Barnsley	Barnsley
E06000050	Cheshire West and Chester	Cheshire
E06000049	Cheshire East	Cheshire
E06000052	Cornwall	Cornwall and Isles of Scilly
E06000053	Isles of Scilly	Cornwall and Isles of Scilly
E09000011	Greenwich	Greenwich
E09000019	Islington	Islington
E07000105	Ashford	Kent
E07000106	Canterbury	Kent
E07000107	Dartford	Kent
E07000108	Dover	Kent
E07000109	Gravesham	Kent
E07000110	Maidstone	Kent
E07000111	Sevenoaks	Kent
E07000112	Shepway	Kent
E07000113	Swale	Kent
E07000114	Thanet	Kent
E07000115	Tonbridge and Malling	Kent
E07000116	Tunbridge Wells	Kent
E08000035	Leeds	Leeds
E09000005	Brent	North West London
E09000009	Ealing	North West London
E09000013	Hammersmith and Fulham	North West London
E09000015	Harrow	North West London
E09000017	Hillingdon	North West London
E09000018	Hounslow	North West London
E09000020	Kensington and Chelsea	North West London
E09000033	Westminster	North West London
E06000027	Torbay	South Devon and Torbay
E07000044	South Hams	South Devon and Torbay
E07000045	Teignbridge	South Devon and Torbay
E08000023	South Tyneside	South Tyneside
E06000033	Southend-on-Sea	Southend
E07000192	Cannock Chase	Stoke and North Staffordshire
E07000194	Lichfield	Stoke and North Staffordshire
E07000195	Newcastle-under-Lyme	Stoke and North Staffordshire
E07000197	Stafford	Stoke and North Staffordshire
E07000198	Staffordshire Moorlands	Stoke and North Staffordshire
E06000021	Stoke-on-Trent	Stoke and North Staffordshire
E07000196	South Staffordshire	Stoke and North Staffordshire
E09000031	Waltham Forest	Waltham Forest and East London and City
E09000025	Newham	Waltham Forest and East London and City
E09000030	Tower Hamlets	Waltham Forest and East London and City
E07000234	Bromsgrove	Worcestershire

For peer review only

E07000235	Malvern Hills	Worcestershire
E07000236	Redditch	Worcestershire
E07000237	Worcester	Worcestershire
E07000238	Wychavon	Worcestershire
E07000239	Wyre Forest	Worcestershire

For peer review only

3. Emergency admission rates for individual local authorities within Pioneers at baseline and follow up, with percentage differences compared to baseline

Local Authority Code	Local Authority	Emergency Admission Rate			Percentage Difference	
		Baseline	2014/15	2015/16	2014/15	2015/16
E08000016	Barnsley	10,992	11,769	12,325	7.07%	12.13%
E06000049	Cheshire East	11,485	12,537	12,954	9.17%	12.80%
E06000050	Cheshire West and Chester	11,033	11,782	11,963	6.79%	8.42%
E06000052/53	Cornwall and Isles of Scilly	8,170	8,061	8,193	-1.33%	0.29%
E09000011	Greenwich	8,168	8,226	9,513	0.71%	16.47%
E09000019	Islington	6,324	6,320	6,096	-0.06%	-3.60%
E07000105	Ashford	8,708	9,785	10,506	12.37%	20.65%
E07000106	Canterbury	9,663	10,503	10,499	8.70%	8.66%
E07000107	Dartford	10,325	12,037	11,063	16.59%	7.15%
E07000108	Dover	9,201	10,411	10,809	13.14%	17.47%
E07000109	Gravesham	9,529	10,825	10,379	13.60%	8.92%
E07000110	Maidstone	9,553	10,470	10,219	9.60%	6.97%
E07000111	Sevenoaks	8,420	8,842	8,114	5.01%	-3.64%
E07000112	Shepway	8,921	9,566	10,274	7.24%	15.17%
E07000113	Swale	10,590	10,746	12,117	1.47%	14.42%
E07000114	Thanet	9,245	9,952	10,194	7.65%	10.27%
E07000115	Tonbridge and Malling	8,842	8,632	8,448	-2.37%	-4.46%
E07000116	Tunbridge Wells	9,188	8,631	7,483	-6.06%	-18.55%
E08000035	Leeds	11,399	9,605	10,155	-15.74%	-10.91%
E09000005	Brent	8,882	8,541	8,425	-3.84%	-5.15%
E09000009	Ealing	10,246	9,828	10,398	-4.08%	1.49%
E09000013	Hammersmith and Fulham	10,312	9,588	9,876	-7.03%	-4.23%
E09000015	Harrow	8,397	8,430	8,084	0.39%	-3.73%
E09000017	Hillingdon	9,753	9,636	9,346	-1.20%	-4.18%
E09000018	Hounslow	8,998	9,751	11,053	8.36%	22.83%
E09000020	Kensington and Chelsea	7,688	7,094	7,048	-7.73%	-8.32%
E09000033	Westminster	7,095	6,454	6,266	-9.04%	-11.70%
E07000044	South Hams	6,591	6,413	6,962	-2.69%	5.63%
E07000045	Teignbridge	7,657	8,182	9,275	6.86%	21.13%
E06000027	Torbay	7,998	8,296	10,173	3.72%	27.19%
E08000023	South Tyneside	11,153	10,445	11,150	-6.35%	-0.03%
E06000033	Southend-on-Sea	9,243	10,397	10,224	12.49%	10.61%
E07000192	Cannock Chase	10,438	10,611	10,083	1.66%	-3.40%
E07000194	Lichfield	8,754	9,424	9,971	7.65%	13.90%
E07000195	Newcastle-under-Lyme	11,919	12,034	12,625	0.96%	5.92%
E07000196	South Staffordshire	8,353	8,907	9,575	6.64%	14.63%
E07000197	Stafford	10,289	10,441	9,900	1.47%	-3.78%
E07000198	Staffordshire Moorlands	8,448	8,649	9,162	2.37%	8.45%
E06000021	Stoke-on-Trent	11,438	11,704	12,959	2.32%	13.29%

For peer review only

E09000025	Newham	7,897	7,535	7,802	-4.58%	-1.20%
E09000030	Tower Hamlets	10,166	9,103	8,795	-10.45%	-13.49%
E09000031	Waltham Forest	9,489	9,333	8,242	-1.64%	-13.14%
E07000234	Bromsgrove	8,452	9,292	9,165	9.94%	8.43%
E07000235	Malvern Hills	8,289	7,937	7,360	-4.25%	-11.21%
E07000236	Redditch	10,556	10,650	11,534	0.89%	9.26%
E07000237	Worcester	9,858	9,180	9,705	-6.88%	-1.56%
E07000238	Wychavon	9,094	8,475	8,760	-6.81%	-3.68%
E07000239	Wyre Forest	7,855	7,366	7,511	-6.23%	-4.38%

4. Sensitivity analysis: excluding Stoke and North Staffordshire Pioneer

	2014/15	2015/16
**Model Coefficients** (p value)		
Intercept	-5.4279 (<0.0001)	-5.4297 (<0.0001)
Non-Pioneer/Pioneer	-0.0205 (0.3009)	-0.0204 (0.3023)
Baseline/Follow-Up	0.0487 (<0.0001)	0.0634 (<0.0001)
Interaction	-0.0302 (0.0489)	-0.0254 (0.1717)
**Percentage Difference** [95% confidence interval]		
Non-Pioneer	4.99 [3.81,6.18]	6.54 [5.21,7.89]
Pioneer	1.86 [-0.85,4.65]	3.87 [0.43,7.42]

Parallel trends interaction term p value = 0.3130

5. Sensitivity analysis: individual baseline years compared to 2014/15

**Baseline Year**	**2010/11**	**2011/12**	**2012/13**
**Parallel Trends P Value**	0.8272	0.3425	0.9235
**Model Coefficients** (p value)			
Intercept	-5.4173 (<0.0001)	-5.4524 (<0.0001)	-5.4144 (<0.0001)
Non-Pioneer/Pioneer	-0.0027 (0.9005)	-0.0034 (0.8652)	-0.0058 (0.7514)
Baseline/Time 2	0.0517 (<0.0001)	0.0522 (<0.0001)	0.0698 (<0.00001)
Interaction	-0.0290 (0.0916)	-0.0285 (0.0730)	-0.0258 (0.0195)
**Percentage Difference** [95% confidence interval]			
Non-Pioneer	5.30 [3.93,6.70]	5.36 [4.09,6.63]	7.23 [6.07,8.40]
Pioneer	2.29 [-0.95,5.64]	2.40 [0.46,5.34]	4.50 [2.48,6.55]

For peer review only - http://bmjopen.bmj.com/site/about/guidelines.xhtml

STROBE Statement—checklist of items that should be included in reports of observational studies

	Item No	Recommendation	Page No
**Title and abstract**	1	(*a*) Indicate the study's design with a commonly used term in the title or the abstract	1
		(*b*) Provide in the abstract an informative and balanced summary of what was done and what was found	2-3
**Introduction**			
Background/rationale	2	Explain the scientific background and rationale for the investigation being reported	4-5
Objectives	3	State specific objectives, including any prespecified hypotheses	5
**Methods**			
Study design	4	Present key elements of study design early in the paper	6
Setting	5	Describe the setting, locations, and relevant dates, including periods of recruitment, exposure, follow-up, and data collection	6-7
Participants	6	(*a*) *Cohort study*—Give the eligibility criteria, and the sources and methods of selection of participants. Describe methods of follow-up<br>*Case-control study*—Give the eligibility criteria, and the sources and methods of case ascertainment and control selection. Give the rationale for the choice of cases and controls<br>*Cross-sectional study*—Give the eligibility criteria, and the sources and methods of selection of participants	6-7
		(*b*) *Cohort study*—For matched studies, give matching criteria and number of exposed and unexposed<br>*Case-control study*—For matched studies, give matching criteria and the number of controls per case	-
Variables	7	Clearly define all outcomes, exposures, predictors, potential confounders, and effect modifiers. Give diagnostic criteria, if applicable	6-7
Data sources/ measurement	8*	For each variable of interest, give sources of data and details of methods of assessment (measurement). Describe comparability of assessment methods if there is more than one group	*6-7*
Bias	9	Describe any efforts to address potential sources of bias	8
Study size	10	Explain how the study size was arrived at	-
Quantitative variables	11	Explain how quantitative variables were handled in the analyses. If applicable, describe which groupings were chosen and why	7-8
Statistical methods	12	(*a*) Describe all statistical methods, including those used to control for confounding	7-8
		(*b*) Describe any methods used to examine subgroups and interactions	-
		(*c*) Explain how missing data were addressed	-
		(*d*) *Cohort study*—If applicable, explain how loss to follow-up was addressed<br>*Case-control study*—If applicable, explain how matching of cases and controls was addressed<br>*Cross-sectional study*—If applicable, describe analytical methods taking account of sampling strategy	-
		(*e*) Describe any sensitivity analyses	8

Continued on next page

For peer review only - http://bmjopen.bmj.com/site/about/guidelines.xhtml

For peer review only

**Results**			
Participants	13*	(a) Report numbers of individuals at each stage of study—eg numbers potentially eligible, examined for eligibility, confirmed eligible, included in the study, completing follow-up, and analysed	9
		(b) Give reasons for non-participation at each stage	-
		(c) Consider use of a flow diagram	-
Descriptive data	14*	(a) Give characteristics of study participants (eg demographic, clinical, social) and information on exposures and potential confounders	9
		(b) Indicate number of participants with missing data for each variable of interest	-
		(c) *Cohort study*—Summarise follow-up time (eg, average and total amount)	-
Outcome data	15*	*Cohort study*—Report numbers of outcome events or summary measures over time	-
		Case-control study—Report numbers in each exposure category, or summary measures of exposure	-
		Cross-sectional study—Report numbers of outcome events or summary measures	*9-10*
Main results	16	(*a*) Give unadjusted estimates and, if applicable, confounder-adjusted estimates and their precision (eg, 95% confidence interval). Make clear which confounders were adjusted for and why they were included	9-11
		(*b*) Report category boundaries when continuous variables were categorized	-
		(*c*) If relevant, consider translating estimates of relative risk into absolute risk for a meaningful time period	-
Other analyses	17	Report other analyses done—eg analyses of subgroups and interactions, and sensitivity analyses	-
**Discussion**			
Key results	18	Summarise key results with reference to study objectives	11
Limitations	19	Discuss limitations of the study, taking into account sources of potential bias or imprecision. Discuss both direction and magnitude of any potential bias	12-13
Interpretation	20	Give a cautious overall interpretation of results considering objectives, limitations, multiplicity of analyses, results from similar studies, and other relevant evidence	12-13
Generalisability	21	Discuss the generalisability (external validity) of the study results	12-14
**Other information**			
Funding	22	Give the source of funding and the role of the funders for the present study and, if applicable, for the original study on which the present article is based	15

*Give information separately for cases and controls in case-control studies and, if applicable, for exposed and unexposed groups in cohort and cross-sectional studies.

Note: An Explanation and Elaboration article discusses each checklist item and gives methodological background and published examples of transparent reporting. The STROBE checklist is best used in conjunction with this article (freely available on the Web sites of PLoS Medicine at http://www.plosmedicine.org/, Annals of Internal Medicine at http://www.annals.org/, and Epidemiology at http://www.epidem.com/). Information on the STROBE Initiative is available at www.strobe-statement.org.

For peer review only
